# Biologically-Inspired Spike-Based Automatic Speech Recognition of Isolated Digits Over a Reproducing Kernel Hilbert Space

**DOI:** 10.3389/fnins.2018.00194

**Published:** 2018-04-03

**Authors:** Kan Li, José C. Príncipe

**Affiliations:** Computational NeuroEngineering Laboratory, Department of Electrical and Computer Engineering, University of Florida, Gainesville, FL, United States

**Keywords:** spike-based learning, noise-robust automatic speech recognition (ASR), keyword spotting, kernel adaptive filtering (KAF), reproducing kernel Hilbert space (RKHS), kernel method, neuromorphic computation

## Abstract

This paper presents a novel real-time dynamic framework for quantifying time-series structure in spoken words using spikes. Audio signals are converted into multi-channel spike trains using a biologically-inspired leaky integrate-and-fire (LIF) spike generator. These spike trains are mapped into a function space of infinite dimension, i.e., a Reproducing Kernel Hilbert Space (RKHS) using point-process kernels, where a state-space model learns the dynamics of the multidimensional spike input using gradient descent learning. This kernelized recurrent system is very parsimonious and achieves the necessary memory depth via feedback of its internal states when trained discriminatively, utilizing the full context of the phoneme sequence. A main advantage of modeling nonlinear dynamics using state-space trajectories in the RKHS is that it imposes no restriction on the relationship between the exogenous input and its internal state. We are free to choose the input representation with an appropriate kernel, and changing the kernel does not impact the system nor the learning algorithm. Moreover, we show that this novel framework can outperform both traditional hidden Markov model (HMM) speech processing as well as neuromorphic implementations based on spiking neural network (SNN), yielding accurate and ultra-low power word spotters. As a proof of concept, we demonstrate its capabilities using the benchmark TI-46 digit corpus for isolated-word automatic speech recognition (ASR) or keyword spotting. Compared to HMM using Mel-frequency cepstral coefficient (MFCC) front-end without time-derivatives, our MFCC-KAARMA offered improved performance. For spike-train front-end, spike-KAARMA also outperformed state-of-the-art SNN solutions. Furthermore, compared to MFCCs, spike trains provided enhanced noise robustness in certain low signal-to-noise ratio (SNR) regime.

## 1. Introduction

Automatic speech recognition (ASR) or the task of translating audio signal into text is an especially challenging problem due to both the non-stationarity of speech signal and the large variations in its spatiotemporal representation. Particularly, the variability in the temporal dimension of speech signal prevents state-of-the-art pattern classifiers such as support vector machines (SVMs) (Scholkopf and Smola, 2001), which are limited to static patterns or fixed (constant) dimension inputs, from being implemented in a straightforward manner. Compounding the issue is that performance often degrades significantly under noisy environments.

**Figure 1:**
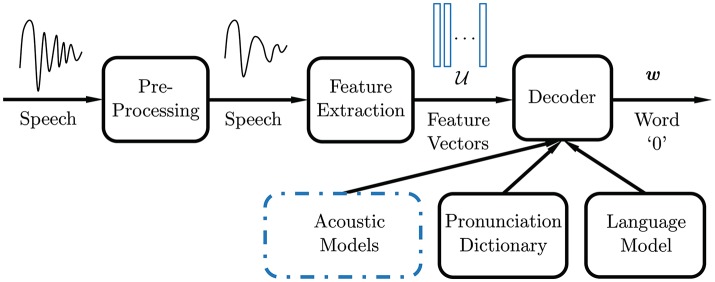
Automatic speech recognition system diagram.

Figure 1 illustrates a typical ASR system. Following pre-processing, which includes speech/non-speech detection and filtering, feature extraction is performed on the post-processed speech signal to form a compact representation. Desirable speech features should emphasize linguistic information over extraneous content such as the speaker's age, emotion, gender, etc. The most commonly used features in speech recognition systems are Mel-frequency cepstral coefficients (MFCCs) (Davis and Mermelstein, 1980). The extraction process involves segmenting the speech signal into quasi-stationary short-time frames of 20–40 ms, overlapped every 10 ms (i.e., frame-rate of 100 fps). For each frame, a Mel-scale filter bank is applied to its power spectrum estimate. The MFCCs are defined as the discrete cosine transform (DCT) of the log energies in the corresponding frequency bands. They measure the power spectrum envelope in each frame, which correlates to the shape of the vocal tract, providing an appropriate representation of the sound or phone being produced.

At the heart of an ASR system is the decoder. Feature vectors are decoded into linguistic units that make up speech, using acoustic models learned from recordings and their corresponding transcripts. Linguistic and pronunciation knowledge are often used to improve the decoding performance (Kuhn and Mori, 1990; Bengio et al., 2003; Mikolov et al., 2010). The standard approach to tackle ASR is to impose a statistical framework by scoring each speech signal with words in a vocabulary on a probability scale, with the most likely word selected as the ASR output. The hidden Markov model (HMM) was the most widely used acoustic model for speech recognition (Rabiner, 1989) until recent years and is still used for many practical applications. Under this statistical framework, the observations or speech feature vectors are modeled as acoustic signals generated by a stationary process, while the transition probabilities in the hidden states account for the time-varying nature of speech. Current advances in accuracy achieved with deep learning (DL) (Hinton et al., 2012) are mismatched with mobile devices and resource-constrained systems, due to difficulty of training, power, and footprint requirements. Conventionally, these applications utilize cloud-based solutions, where processing is performed on large remote servers. However, this imposes additional demands on quality of service. There are many mobile applications where the on-device acoustic model output accuracy is insufficient.

**Figure 2:**
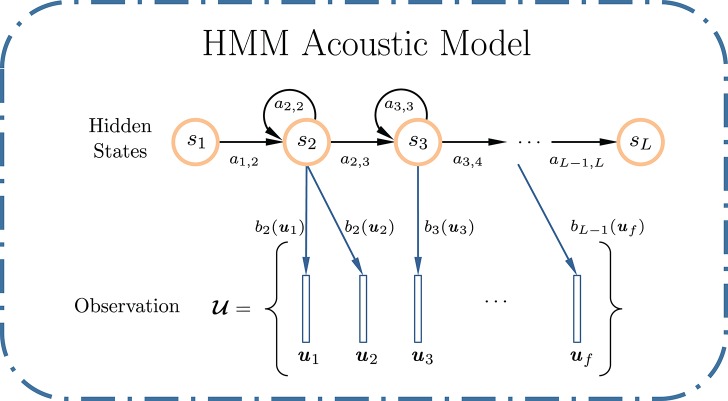
Example of an *L*-state left-to-right discrete HMM used for ASR, with two non-emitting states: *s*_1_ and *s*_*L*_. For each emitting state, the HMM can only remain in the same state or move to the next state on its right.

Figure 2 shows a typical discrete HMM, parametrized by an initial state distribution π = {π_*i*_ = Pr(*S*_1_ = *s*_*i*_)}, a state transition probability matrix **A** = {*a*_*i,j*_ = Pr(*S*_*t*_ = *s*_*j*_|*S*_*t*−1_ = *s*_*i*_)}, an observation distribution **B** = {*b*_*i*_(**u**_*t*_) = Pr(**u**_*t*_|*S*_*t*_ = *s*_*i*_)}, where $U=\left\{{\text{u}}_{1},{\text{u}}_{2},\cdots ,{\text{u}}_{f}\right\}$ is an *f*-frame observation sequence, and *S* = {*s*_1_, *s*_2_, …, *s*_*L*_} is the underlying state sequence of length *L*, which forms a first-order Markov chain. The Gaussian mixture model (GMM) is typically used to approximate the observation distribution **B**. An HMM (π, **A**, **B**) can be estimated using the Baum-Welch (BW) algorithm (Baum et al., 1970), a special case of the expectation-maximization (EM) algorithm (Dempster et al., 1977). In ASR, one HMM is trained for each speech unit (e.g., phone, syllable, word, etc.,) in the vocabulary. A test utterance is compared to all trained HMMs, in order to determine the likelihood that it was generated by a particular HMM. This framework represents an unsupervised learning paradigm. As a maximum-likelihood estimation (MLE) method, it relies on strong assumptions on the statistical properties of the observed phenomenon, but lacks discriminative power among different models.

Since humans naturally and very efficiently decode speech and perform better than most ASR systems, especially in noisy environments, it is only logical for researchers to turn to biological inspiration in the design of ASR systems. As a matter of fact, MFCC already makes use of the psychoacoustic properties of the auditory system (the Mel scale imitates the cochlea by employing linearly and logarithmically distributed filters along the frequency axis, with the cutoff at 1 kHz), a fine tuned preprocessing step in the human auditory system. The pressure waves originating from the cochlea are translated into spike trains by the peripheral auditory neurons, which travel through nerve fibers to the auditory cortex. The computation in this complex and hierarchical structure is carried out via action potential timing information. Computing with spikes is therefore an important aspect to bio-inspired ASR.

There has been limited research in spike train representation for spoken word recognition (Hopfield and Brody, 2001; Verstraeten et al., 2005; Wade et al., 2010; Zhang et al., 2015). The state-of-the-art spike-based ASR systems are based on spiking neural network (SNN) such as liquid state machines (LSMs) (Maass et al., 2002). LSM utilizes a large randomly initialized network with recurrent connections, also referred to as a dynamic reservoir or liquid. The parameters of the liquid remain fixed, and only a readout layer is adapted through training to optimally project the network or liquid states onto the desired output. The LSM falls under a general framework called reservoir computing (RC), which is further identified as an echo state network (ESN) (Jaeger, 2001) for continuous valued inputs and LSM for spike train inputs. The primary advantage of the LSM approach is that it does not require consideration for time dependency of the learning task, since all temporal processing is performed implicitly in the recurrent neural circuit. RC is free from the problems associated with gradient-based recurrent neural networks training such as local optima, slow convergence, and high computational complexity. However, performance depends largely on the reservoir hyperparameters that need to be cross-validated appropriately to find an optimal solution, without which RC is a less reliable convex universal learning machine (CULM) than conventional adaptive networks using kernel adaptive filtering (Príncipe and Chen, 2015). Furthermore, producing a constant output for time-varying liquid state is a major challenge for LSM, since its memory-less readout has to transform the transient and non-stationary states of the liquid into a stable output without the assistance of stable states or attractors (Maass et al., 2002).

In our previous work (Li and Príncipe, 2016), we introduced a novel online kernel adaptive filtering algorithm: the kernel adaptive autoregressive-moving-average (ARMA) or KAARMA. We demonstrated this kernelized recurrent network's ability to model dynamical systems and as a bit-stream classifier using the benchmark Tomita grammars. Specifically, we showed that KAARMA-based solutions can outperform LSMs on spike data, which opened the door for many novel neuroscience applications (Dura-Bernal et al., 2016). Furthermore, we have successfully applied the methods to model flight dynamics of insects and plant growth patterns (Li and Príncipe, 2017a,b; Li et al., 2017). Since speech production is both nonlinear and non-stationary in nature, KAARMA can deliver computationally efficient solutions for ASR as we demonstrate below.

In this paper, we propose a novel paradigm to work with spike timing information. Instead of projecting the input spike train nonlinearly into a much higher dimensional space using a recurrent interconnection of spiking neurons as is done with LSM, we project the input spike trains into an infinite dimensional function space (RKHS) using positive definite functions, where we train a linear state-space model with a very small order using backpropagation and the kernel trick. The theory of adaptive signal processing is greatly enhanced through the integration with the theory of RKHS. By performing classical linear methods in an infinite-dimensional feature space, online kernel learning (Kivinen et al., 2004), such as kernel-Adaline (Frieß and Harrison, 1999), kernel recursive least-squares (KRLS) (Engel et al., 2004), kernel least mean square (Liu et al., 2008), and extended-KRLS (Liu et al., 2009) algorithms provide general nonlinear solutions in the original input space. It also gives rise to kernel Kalman implementations, such as using subspace kernel principal component analysis (Ralaivola and d'Alche Buc, 2005) and statistical embedding (Zhu et al., 2014) to model nonlinear dynamics.

A major advantage of the KAARMA algorithm is that it works with functions in the RKHS and changing the kernel function does not impact the underlying learning algorithm. Therefore, KAARMA is agnostic to the type of input and can be applied to static data using Gaussian kernels, or directly to spike trains, by designing an appropriate spike kernel (Park et al., 2012, 2013). In this paper we use a biologically-inspired auditory filterbank and a LIF neuron model to convert the continuous-amplitude signal output from each channel of the filterbank into a sparse spike train representation, to create a multichannel spike train, encoding the signal-structure changes in each frequency band. The spike trains are then segmented using a sliding window into frames of fixed duration and frame rate or stride, similar to conventional speech processing. A special designed temporal kernel then maps the spike-train frames to an RKHS by estimating the distance between successive frames of the spike trains, using their precise spike timings. Inference is performed not on individual frames, but on sequences of spike-train frames without assumption on the sequence length. Nonlinear ARMA networks have theoretical capability to model dynamics of arbitrary complexity. This methodology suggests a novel way to apply spike-based computation using a recurrent neural coding algorithm in RKHS as an alternative for a biologically-inspired robust ASR system. Without any feature engineering step, we evaluate how well this spike-based KAARMA ASR performs compared to conventional amplitude-based MFCC-KAARMA and other SNN solutions. We also evaluate the inherent noise-robustness of the spike-train sparse representation, due to the smoothing effect of the integration operation in the LIF neuronal model.

The rest of this paper is organized as follows. In section 2, we briefly introduce the KAARMA methodology. We present its application for bio-inspired spike-based ASR in section 3. Performances of the proposed KAARMA classifiers are evaluated in section 4. Section 5 concludes this paper.

## 2. Methods

We would like to model and learn the temporal evolution of speech time-series acoustical features' structure encoded in spike trains. The goal, here, is a bio-inspired ASR system where as much of the traditional speech pipeline as possible is replaced by a recurrent network architecture. Specifically, we wish to evaluate an end-to-end spike-based keyword spotting system, without hand-designed feature extraction algorithm, past the spike-generation stage. Furthermore, we wish to use a unifying framework that does not depend on input signal type. For example, conventional artificial neural network and SNN have completely different output and learning mechanisms due to the non-differentiable activation functions associated with discrete spikes. To accomplish this, we apply the theory of RKHS to map the inputs into a function space and construct a recurrent network in this space. This way, the learning algorithm is defined not in terms of the input representation (continuous-valued attributes vs. discrete spikes), but in terms of dot products between respective infinite-dimensional features, where they can be computed in closed form using the kernel trick. Thus, we are free to choose the input representation independently with an appropriate reproducing kernel, and changing the input-kernel pair does not impact the learning algorithm itself. An additional drawback of conventional speech pipeline is alignment, specifically frame-level training targets. We can resolve all the issues mentioned by modeling speech as a dynamical system and treating isolated word recognition as a grammatical inference task trained on sequences and not on individual frames, using the kernel adaptive ARMA algorithm.

### 2.1. Kernel adaptive ARMA algorithm

Here, we briefly introduce the KAARMA algorithm for isolated-word speech recognition or keyword spotting, while the adaptation of parameters is presented in the Appendix (see Supplementary Material) for completeness. For a more in-depth derivation, please refer to Li and Príncipe (2016).

A dynamical system approach studies the evolution of observables over time according to specific rules. We can trace it to a classical Newtonian root: the forces are much simpler to describe than planetary motions. Under this framework, even seemingly-chaotic time series actually follow an easy to explain hidden order, and a dynamical model allows us to find such attracting behavior. Rule discovery provides a compact and convenient way to analyze and model a class of equivalent trajectories but with large variations in realization.

First, let us define a dynamical system using a state-space representation with a general continuous nonlinear state-transition function **g**(·, ·) and an observation function **h**(·) :

(1) $$x_{i}=g(s_{i-1},u_{i}),$$

(2) $$y_{i}=h(x_{i})\overset{\Delta }{=}h\circ g(s_{i-1},u_{i}),$$

with input vector ${\text{u}}_{i}\in {\mathbb{R}}^{n_{u}}$, hidden state vector ${\text{x}}_{i}\in {\mathbb{R}}^{n_{x}}$, output vector ${\text{y}}_{i}\in {\mathbb{R}}^{n_{y}}$, the augmented state vector ${\text{s}}_{i}\overset{\Delta }{=}{\left[{\text{x}}_{i},{\text{y}}_{i}\right]}^{T}$, and the function composition operator ◦. For our application, the state-transition function **g**(·, ·) describes the dynamics driven by the input speech **u**_*i*_ and the previous state (for isolated word, all speech sequences are assumed to have the same initial state). The sequence output **y**_*i*_ is related to the states and inputs by observation function **h**(·).

Using a grammatical-inference formulation, the only thing we know during training are labels for the full sequences or speech utterances, i.e., the final sequence output **y**_*f*_ = {±1} for positive or negative examples of a target class or word model. The state and transition functions can be parametrized with weight values of a fully connected recurrent network and learned using backpropagation of the label error at the end of each speech sequence. This task is an inference problem as opposed to a prediction one, i.e., a sequence-based approach vs. the conventional frame-based approach of an HMM. There is no prediction of the next frame of speech in the utterance sequence. The network either accept or reject an entire utterance at the end of each sequence. This is a more difficult problem than prediction, since we do not have complete classification knowledge of every subsequence (i.e., when prediction and inference are equivalent). On the other hand, it does not require a frame-level target or alignment, i.e., a desired signal **d**_*i*_ is not required at each time/frame index of output **y**_*i*_, only for the final index **y**_*f*_; the internal state trajectories **s**_*i*_ are also learned directly from the training sequences (given a fixed initialized state) without any observables except at the end of the sequence when **y**_*f*_ = {±1} for ${\text{s}}_{f}={\left[{\text{x}}_{f},{\text{y}}_{f}\right]}^{T}$; and, this dynamical model makes no assumption on the speech utterance duration or sequence length *f*, i.e., it can operate on sequences of arbitrary length.

Adaptation of parameters in the linear state model is very well understood, and the famed Kalman filter (Kalman, 1960) presents a very efficient recursive update algorithm that can be computed in real time. The problem of the linear state model is that it is not universal, i.e., it only can solve problems with small error when the desired response exists in the span of the input space (Haykin, 1998). Past work with dynamical modeling of speech shows that the linear dynamical model is not competitive with the HMM statistical model. The theory of RKHS allows classical linear method to produce general nonlinear solutions, and by operating in a new, function space, we are freed from the limitations of the original input representation/space.

To emphasize the input-agnostic property of a function-space formulation for applications using either continuous-valued input or discrete-time events, we first describe the KAARMA algorithm using a generic input sequence **u**_*i*_, then specify it for spikes in section 2.2, which basically amounts to a simple substitution on the kernel choice. Using the representer theorem, we can express the state-space model Equation (1-2) as a set of weights (functions in the input space) in the joint RKHS $H_{su}\overset{\Delta }{=}H_{s}⊗H_{u}$

(3) $$\Omega \overset{\Delta }{=}\Omega_{H_{su}}\overset{\Delta }{=}\left[\begin{array}{c} g(\cdot ,\cdot )\\ h\circ g(\cdot ,\cdot ) \end{array}\right],$$

where ⊗ is the tensor-product operator. Finally, the kernelized state-space model becomes

(4) $$s_{i}=\Omega^{T}\psi (s_{i-1},u_{i}),$$

(5) $$y_{i}=Is_{i},$$

where $\psi \left({\text{s}}_{i-1},{\text{u}}_{i}\right)\overset{\Delta }{=}\varphi \left({\text{s}}_{i-1}\right)⊗\phi \left({\text{u}}_{i}\right)$ is a feature in the joint RKHS and $𝕀\overset{\Delta }{=}\left[0\;I_{n_{y}}\right]$ is a fixed selector matrix with **I**_*n*_*y*__ is an *n*_*y*_ × *n*_*y*_ identity matrix, used to extract the output components **y** from the augmented state vector **s**. This is analogous to a second-order recurrent neural network defined in a function space in our previous work (Li and Príncipe, 2016).

It follows that the tensor-product kernel is defined as

(6) $$\begin{array}{l} {\langle \psi (s,u),\psi (s^{\prime },u^{\prime })\rangle }_{H_{su}}=K_{su}(s,u,s^{\prime },u^{\prime })=(K_{s}⊗K_{u})(s,u,s^{\prime },u^{\prime })\\ \;=K_{s}(s,s^{\prime })\cdot K_{u}(u,u^{\prime }). \end{array}$$

This construction has several advantages over the simple concatenation of the input **u** and the state **s**. First, the product of two positive-definite (PD) kernels is also a PD kernel. Second, since learning is performed in an RKHS using features, there is no constraint on the original input signal representation or the number of signals, as long as we use an appropriate reproducing kernel for each signal. Additionally, the sum or average of two PD kernels is also a PD kernel for multi-channel input. More importantly, this formulation imposes no restriction on the relationship between the signals in the original input space. This is especially useful for input signals having different representations and spatiotemporal scales. Specifically, under this framework, we can model a neurobiological system, taking continuous-amplitude local field potentials, discrete-events-in-continuous-time spike trains, and vectorized state variables as inputs.

**Figure 3:**
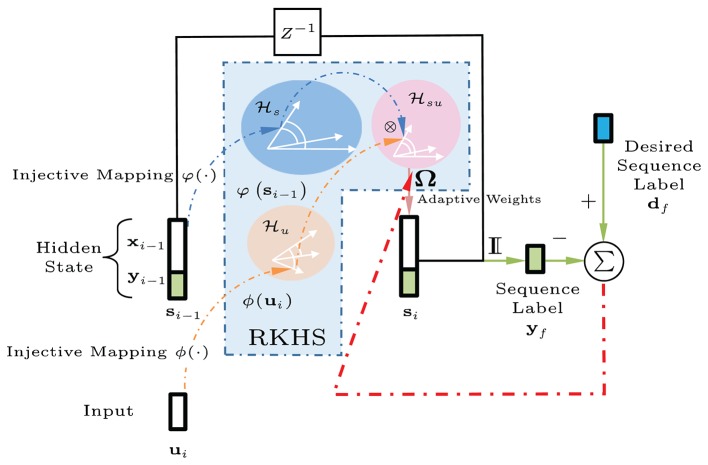
Block diagram of the kernel adaptive ARMA (KAARMA) algorithm. The values of the adaptive weights Ω in the feature space are learned using backpropagation and the kernel trick. In general, the states ***s***_*i*_ are assumed hidden, and during training, the desired value for label **y**_*i*_ is only observed at the end of the sequence, i.e., at the final indexed time step *i* = *f*.

Figure 3 shows a graphical interpretation of a dynamical system defined in a joint RKHS using a product kernel. Data instances are processed using inner products or similarity measures. The tensor-product kernel is analogous to a soft-valued logical AND operator on the joint similarity measure. To output a desired next state requires both an appropriate current input AND the right previous state. In general, the states **s**_*i*_ are assumed hidden, and during training, the desired signal does not need to be available at every time step, e.g., a deferred desired output value (±1 sequence label vector) for **y**_*i*_ may only be observed at the final indexed step *i* = *f*.

The KAARMA preserves the simplicity of linear dynamical models with the universality of functional spaces, so it is an attractive candidate to substitute linear dynamical systems in computational neuroscience applications using either local field potentials or spike trains. In computational neuroscience there is a chasm between the methodologies for spike trains and continuous amplitude signals that can be easily bridged with RKHS methodologies. Indeed the same machine learning code can be utilized for both types of signals, once specific kernel are designed for each signal modality. The application for speech recognition exemplifies a statistical learning approach to work with spike trains, which improves the biorealism of the processing and lets us take advantage of the spike timing information.

The fundamental building block for designing the KAAMA for spike trains is therefore the kernel, which will be explained next.

### 2.2. Reproducing kernel hilbert space (RKHS) for spike trains

**Figure 4:**
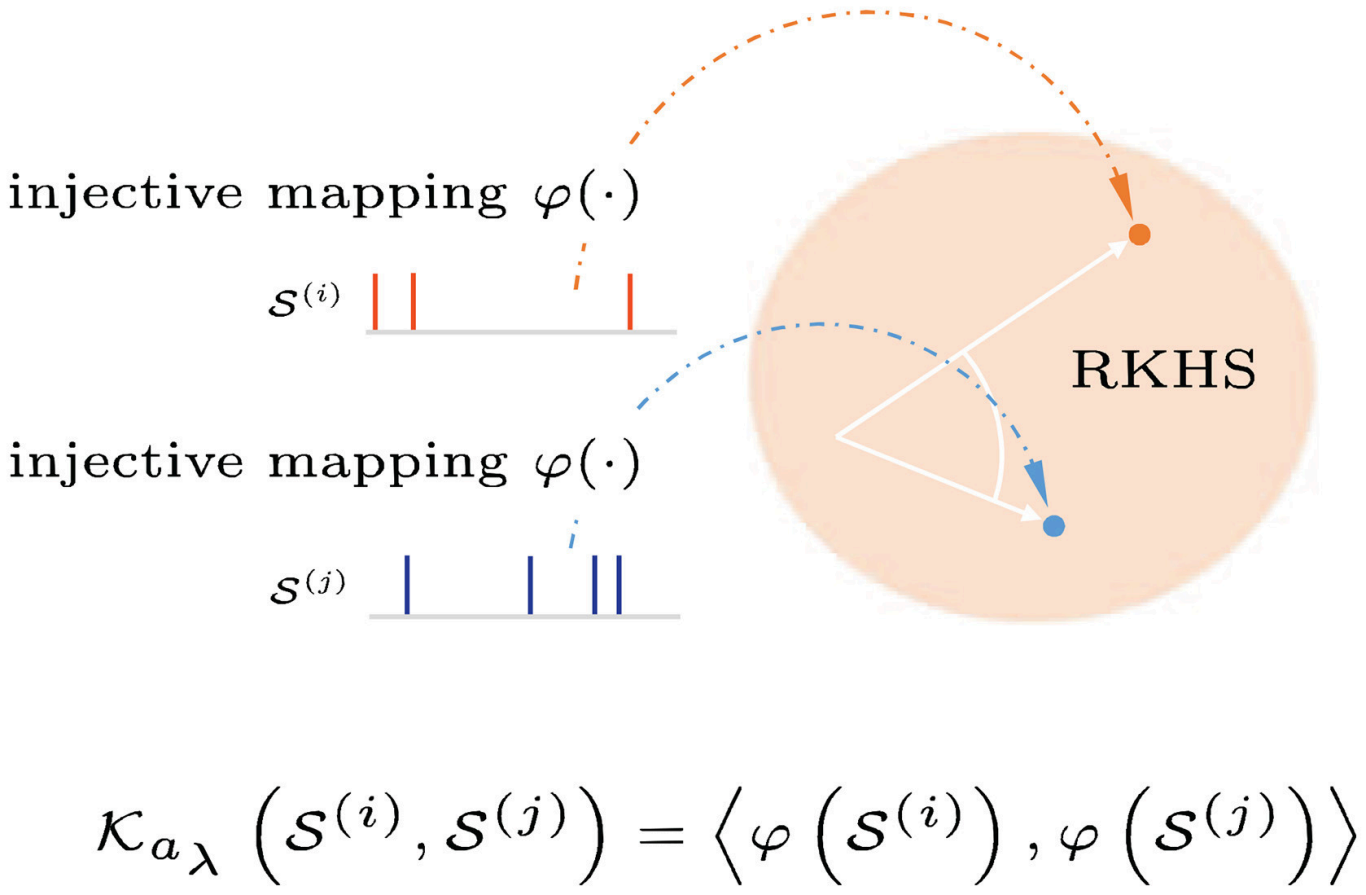
Graphical interpretation of a reproducing kernel Hilbert space defined on spike trains. Spike trains with precise spike timings are mapped into an infinite-dimensional feature space (Hilbert space). Applying the kernel trick allows inner products in this space to be computed without explicit reference to the feature representation.

We want to study how information is represented and processed as spike trains using the theory of RKHS. Since spike trains are devoided of a natural algebra, they impose many challenges to signal processing methods. We must first establish a space for computation or transformation to a space with the necessary properties. The approach explained here is to define a proper kernel function on spike trains to capture non-parametrically the instantaneous temporal structure and the variability of the spike trains of interest. Once a positive-definite kernel is defined, it maps the spike trains into a Hilbert space of functions which allows signal processing tools to be applied directly through the kernel trick, as shown in Figure 4.

We use the Schoenberg kernel (Park et al., 2012), a universal binless nonlinear spike train kernel, to define the joint tensor-product RKHS. This kernel is bio-inspired using conditional intensity function of a temporal point process. Among spike train kernels [count and binned kernels, spikernel (Shpigelman et al., 2005), linear functional kernels (Paiva et al., 2009), and nonlinear functional kernels (Park et al., 2012)], the Schoenberg kernel has three distinct advantages: (1) provides injective mapping, (2) embeds arbitrary stochasticity of neural responses as the sample mean in the RKHS, and (3) approximates arbitrary function on spike trains as a universal kernel (Park et al., 2013).

A spike train or sequence of *M* ordered spike times, i.e., $S^{\left(i\right)}=\left\{t_{m}\in T:m=1,\cdots ,M\right\}$ in the interval T=[0,T], can be viewed as a realization of an underlying stochastic point process with conditional intensity function $\lambda \left(t|H_{t}^{\left(i\right)}\right)$, where t∈T=[0,T] denotes the time coordinate, and $H_{t}^{\left(i\right)}$ is the history of the process up to time *t*. The point process is approximated as a zero-baseline-rate Hawkes process (Hawkes, 1971). Schoenberg kernel between the conditional intensity functions of two point processes (Paiva et al., 2009; Park et al., 2012; Dura-Bernal et al., 2016) is defined as

(7) $$K_{a_{\lambda }}(\lambda (t|H_{t}^{\left(i\right)}),\lambda (t|H_{t}^{\left(j\right)}))\overset{\Delta }{=}\exp\left(-a_{\lambda }\int_{\tau }{\left(\lambda (t|H_{t}^{\left(i\right)})-\lambda (t|H_{t}^{\left(j\right)})\right)}^{2}dt\right),$$

where *a*_λ_ > 0 is the spike-train kernel parameter. The conditional intensity function of the self-exciting point process with zero background rate is approximated by convolving the precise spike times with a smoothing kernel *g*(*t*), yielding

(8) $$\hat{\lambda }(t)=\sum_{m=1}^{M}g(t-t_{m}),\{t_{m}\in T:m=1,\cdots ,M\}.$$

**Figure 5:**
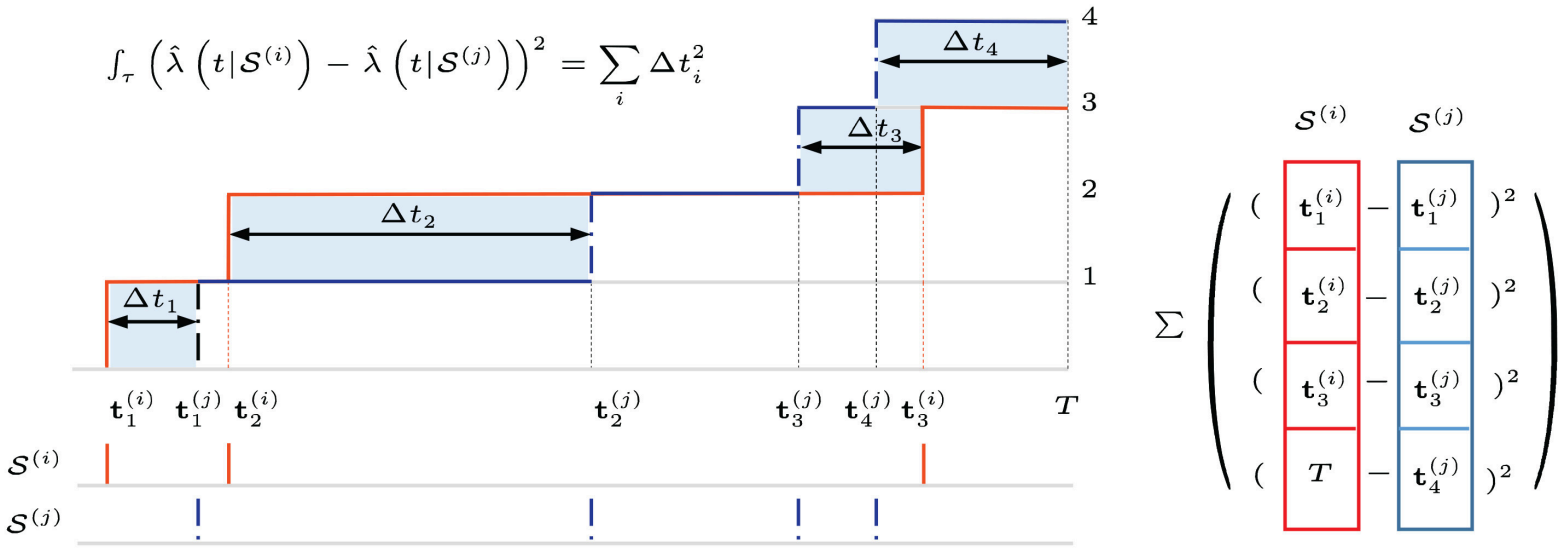
The Schoenberg spike kernel computes the similarity between a pair of spike trains. In this application, we compare the conditional intensity function estimates for spike-train frames $S^{\left(i\right)}$ and $S^{\left(j\right)}$ at different times in a given frequency band or channel. Using Heaviside step function for smoothing greatly simplifies the computation. We can visualize it as a sum of squared pair-wise spike-timing differences between two unit-step staircase functions (squared areas in blue) or as squared Euclidean distance on ordered sets of spike timings, with the fewer-spike set padded with frame duration time *T*. For multichannel spike input, the sum or average distance is used.

It computes the similarity between a pair of spike trains in T, either from a single neuron at different times or from a pair of neurons. In this application, instead of two spike trains from different frequency bands, we are interested in quantifying the time-series structure or difference in conditional intensity functions across time of the same spike channel. For computational simplicity, we use the rectangular function $g\left(t\right)=\frac{1}{𝔗}\left(U\left(t\right)-U\left(t-T\right)\right)$, where *U*(*t*) is a Heaviside function and T is chosen to be much greater than the average inter-spike interval. Since we are interested in time-binned or frame-based raw spike events, T is effectively set to the frame duration. Figure 5 illustrates this squared distance between the conditional intensity function estimates of two spike-train frames $S^{\left(i\right)}$ and $S^{\left(j\right)}$ at different times for a given frequency band or channel, i.e., the integral in Equation (7) using Equation (8). In this formulation, the spike-train distance only depends on the precise spike timings in ordered sets. When two spike trains are “close,” more of their spike timings are synchronized, yielding a smaller pair-wise distance.

**Figure 6:**
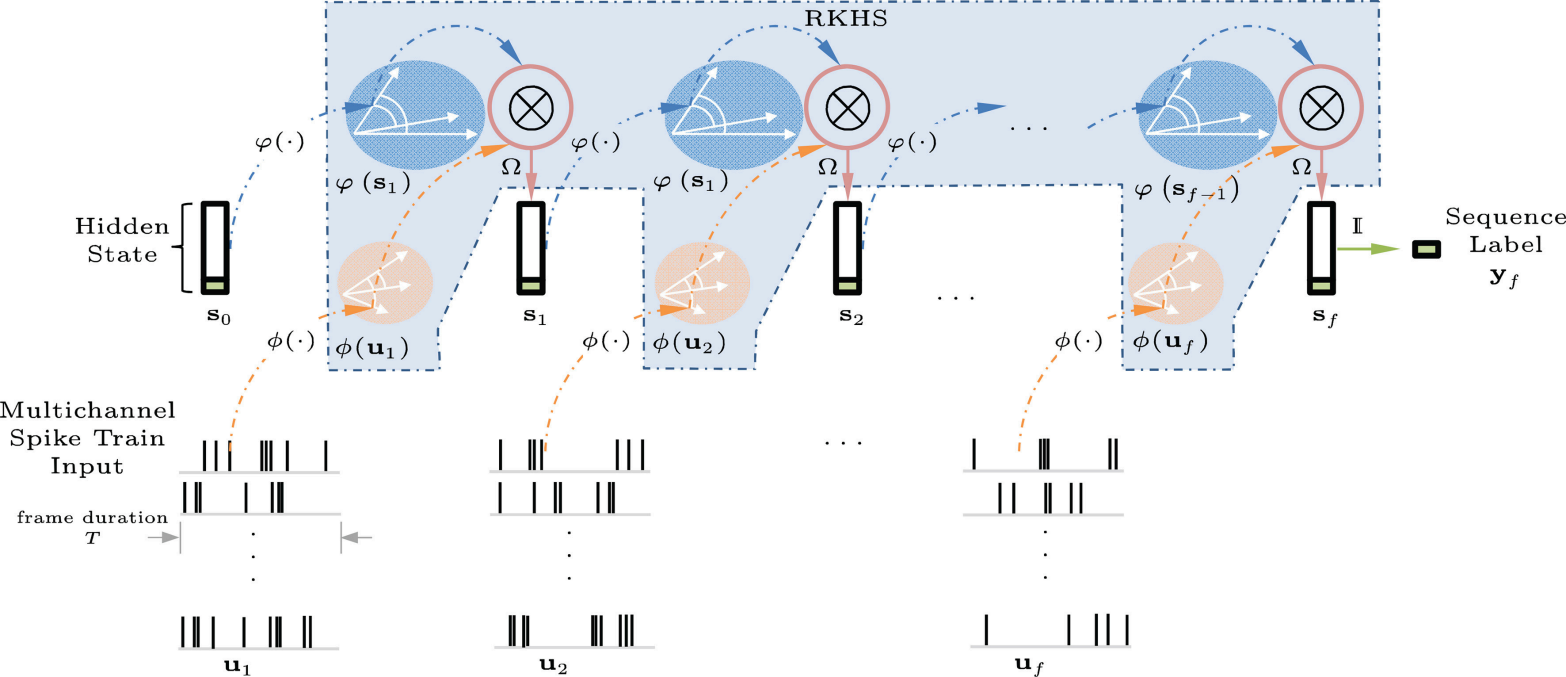
Spike-input KAARMA network unfolded in time for *f* frames. The multichannel spike-train input frames are mapped into a joint RKHS with the current hidden state vector using a tensor-product kernel to generate the next state vector. The final state vector at frame *f* contains the prediction label for the entire sequence.

For multichannel spike input, we sum or average the spike-train distances over all channels in each time frame. Specifically, the multichannel spike trains are segmented into frames or smaller spike trains the same way as the MFCCs, with a frame duration of 25 ms and rate of 100 fps. Figure 6 illustrates a KAARMA network working directly on spike trains.

### 2.3. Comparisons between spike-based kernel approach vs. LSM

The LSM and the KAARMA are both adaptive recurrent models that operate with spike trains, but the similarity ends here.

The LSM uses a recurrent layer of spiking neurons, designed by a user, to project the input spike data into a high dimensional space, where it will be easier to find a learned projection that fulfills the data processing goal. Clearly, not all projections to high dimensional spaces will preserve the information contained in the input spike train, therefore, the designer must select a hyperparameter that achieves the prescribed separation property or SP (Maass et al., 2002). SP is quantified by a kernel-quality measure proposed in Maass et al. (2005) that is based on the rank of a matrix formed by the system states corresponding to different input signals (Bertschinger and Natschläger, 2004). Therefore, SP is signal and application dependent, which means that creating the optimal liquid is still today more of an art than a science. The advantage of the LSM is that it uses directly the instantaneous intensity function of the spike trains because it is a dynamical system.

The KAARMA handles the processing of spike trains in a very different way. First, the spike trains are projected to an infinite dimensional space of functions (RKHS) with the Schoenberg kernel using the instantaneous conditional intensity function estimated on an interval. Linear models in RKHS are universal mappers, i.e., they can approximate any input-output map. In this space, one can train a linear state model directly from data to learn the spike train structure and deliver a high quality mapping with very small model orders, using directly the input data (the representer theorem). So instead of a high dimensional and usually randomly created and fixed reservoir that an LSM uses, the KAARMA uses the functions in the Hilbert space centered by the projected input spike trains. This RKHS is based entirely on the available data samples with optimized adaptive weights. The spike kernel still operates with instantaneous information but now in the conditional intensity function of learned data, which is a suitable approximation to the intensity function, but requires the selection of a hyperparameter.

## 3. Automatic speech recognition system using KAARMA

We can treat certain speech recognition tasks as grammatical inference problems and apply the KAARMA algorithm to learn temporal structures of speech features with arbitrary length, analogous to syntactic pattern recognition involving the Tomita grammars (Li and Príncipe, 2016). As a recurrent network, the KAARMA algorithm exploits the full contextual information of the entire feature sequence to create a discriminative model. It makes no assumption on the model topology of the data, and the states are learned completely from the observations.

Many spoken words share similar or identical acoustic features. Given the large variations in speech production, common trailing phoneme can be difficult for recurrent systems learning long-term dependencies, where long-drawn-out overlapping ending sequences can cause two different word models to converge. One simple way to circumvent this problem, without significant change to the experiment, is to simply reverse the temporal order of the acoustic features, such that the trailing sequences no longer overlap, and train a KAARMA classifier that recognizes this new input ordering. Digits that used to share the same trailing phoneme may end up in different ones (of course the opposite can also happen). To maximize recognition rate for each digit, we can combine the results of two networks trained on sequences in the natural left-to-right temporal direction and the reversed right-to-left ordering, by simply multiplying their softmax scores. Flipping the sequence ordering generates a new complementary grammar that can be combined to enhance classification results. This is a feature that is entirely missing in HMMs, due to the Markov property that states are formed locally and only operate on adjacent observation vectors. States in a recurrent network, on the other hand, are memory units which encode the entire past history, starting from an initial state, and indicate a global status. To further reduce the need to learn long-term dependencies and to simplify computation, we can partition a speech feature sequence into smaller segments, without the need for complicated alignment, which we discuss in detail next.

### 3.1. KAARMA chain

Here we formulate the KAARMA chain approach for isolated word recognition under a simple statistical framework. First, let us revisit the conventional HMM in Figure 2. In the hidden Markov model, speech signal, specifically, the sequence of acoustic feature vectors $U=\left\{{\text{u}}_{1},{\text{u}}_{2},\cdots ,{\text{u}}_{f}\right\}$ is generated by a finite state automaton consists of *L* states *S* = {*s*_1_, *s*_2_, …, *s*_*L*_} under a probabilistic framework. An HMM is equivalent to a stochastic regular grammar (Lari and Young, 1990). Each speech unit is associated with a specific Markov model *M*_*i*_ comprised of states from *S* according to a predefined topology. The left-to-right (Bakis) model is the most commonly used topology for speech recognition (Bakis, 1976). States are aligned from left to right to form a single Markov chain, indexed incrementally and with only self- or right-transitions allowed, i.e., *a*_*i,j*_ = 0, for *j* < *i*. Furthermore, the initial state is fixed at state *s*_1_. Left-to-right HMMs are able to model the temporal properties of speech.

The training and recognition criteria for HMMs are based on maximizing the *a posteriori* probability $\text{Pr}\left(M_{i}|U\right)$ that the observation U has been produced by the HMM *M*_*i*_. Using Bayes' rule, we can rewrite the expression as

(9) $$\text{Pr}(M_{i}|U)=\frac{\text{Pr}(U|M_{i})\text{Pr}(M_{i})}{\text{Pr}(U)},$$

where $\text{Pr}\left(U|M_{i}\right)$ is the maximum likelihood estimate (MLE) criterion, Pr(U) is constant during recognition, and the *a priori* probability Pr(*M*_*i*_) is an appropriate language model.

The BW algorithm can be used to maximize the likelihood estimate of the parameters of a HMM, given the set of observed feature vectors. Alternatively, the MLE can be replaced by the Viterbi criterion, where only the most probable state sequence of producing U is considered

(10) $$\hat{\text{Pr}}(U|M_{i})=\underset{S}{\max}\text{Pr}(S,U|M_{i}),$$

and the optimal S^*^ is given by

(11) $$S^{*}=\underset{{}_{S}}{\arg\max}\prod_{\ell =1}^{L}\text{Pr}(s_{\ell }|s_{\ell -1})\text{Pr}(u_{\ell }|s_{\ell }),$$

which can be solved using the Viterbi algorithm (Viterbi, 1967). This frame-based approach is fundamentally different from our novel sequence-based approach which requires no alignment or frame-level target for isolated word recognition.

Under a hybrid ANN-HMM paradigm, connectionist statistical methods (Franzini et al., 1990; Levin, 1990; Morgan and Bourland, 1990; Niles and Silverman, 1990; Robinson, 1994) were proposed as improvements to the standard HMM. It is well-established that the outputs of a multilayer perceptron (MLP) operating in classification mode can be interpreted as estimates of the local *a posteriori* probabilities of output classes conditioned on the input (Bourlard and Wellekens, 1990)

(12) $$y_{j}^{*}(u_{i})=p(s_{j}|u_{i}),$$

where $y_{j}^{*}$ is the optimal (MLP with sufficient parameters and no local minimum) classification output value for state *s*_*j*_. In the hybrid approach the *a posteriori* probabilities are converted into the HMM emission probabilities *p*(***u***_*i*_|*s*_*j*_) by dividing the MLP output by the prior class probabilities. To provide context information, 2*c* + 1 frames were used at the input (where *c* is the context window parameter, with the current input frame centered in the middle) of the MLPs in Boulard and Morgan (1993), and RNNs were used in Robinson (1994).

A mixture-of-experts ESN architecture with a winner-take-all update strategy exhibited superior noise-robustness than HMM (Skowronski and Harris, 2007) for continuous-valued human factor cepstral coefficients (HFCC) (Skowronski and Harris, 2004). Multiple readout filters are grouped together to form a state (paralleling the Gaussian mixture of a Bakis HMM state), and test utterances were classified as the word model with the lowest mean-squared prediction error (MSE) along the Viterbi path for each model. Context features were used (first- and second-order temporal derivatives over ±4 frames), along with the log energy of each frame. Our approach, on the other hand, learns the contextual information directly from the input stream, without being hard-coded at each time step (a 12-dimension vector vs. the 39-dimension speech feature of the ESN), and the internal states are integrated under a unifying framework. The KAARMA recognition results are also directly obtained, without the need for Viterbi computation. Furthermore, while the ESN matched the baseline HMM performance for noise-free conditions, we will show that automatically learned recurrency can outperform HMM using the same inputs, for a computationally simpler implementation.

#### 3.1.1. Grammar states

Instead of using universal approximators as local state emission probability estimators in the HMM framework, we can solve the statistical recognition criterion directly using the KAARMA algorithm. Recall that the MAP is defined as

(13) $$M^{*}=\underset{{}_{M}}{\arg\max}\text{Pr}(M|U),$$

where *M* is the inference model, which is equivalent to maximizing the *a posterior* state sequence or most probable state sequence for each model.

**Figure 7:**
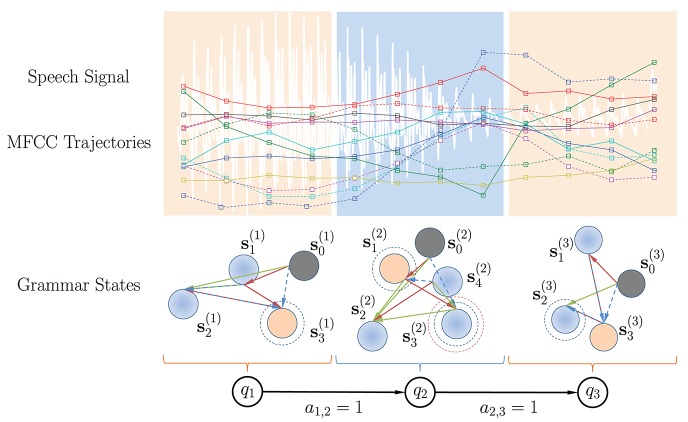
Example of a KAARMA chain of three equal-partition grammar states.

Let us define the states in a KAARMA chain as context-free grammars, denoted by $Q=\left\{q_{1},q_{2},\cdots ,q_{L}\right\}$. This distinction is made to not confuse a grammar state *q*_*i*_ with the KAARMA internal hidden-state variables **s**_*i*_ (grammar state *q* is a discrete set and network hidden state **s** is a vector). Each grammar state *q*_*i*_ has its own set of unique internal hidden-states **s**^(*i*)^ that transition according to the rules learned directly from data, i.e., $q_{i}=\left\{{\text{s}}_{0}^{\left(i\right)},{\text{s}}_{1}^{\left(i\right)},\cdots ,{\text{s}}_{n_{i}-1}^{\left(i\right)}\right\}$. Under this formulation, a single KAARMA network (global grammar with $Q=\left\{q_{1}\right\}$) trained on the entire observation trajectory $U=\left\{{\text{u}}_{1},{\text{u}}_{2},\cdots ,{\text{u}}_{f}\right\}$ can be viewed as an HMM with only a single state, e.g.,

(14) $${\tilde{y}}_{f}^{\left(i\right)}=\frac{\exp(y_{f}^{\left(i\right)})}{\sum_{j=0}^{9}\exp\left(y_{f}^{\left(j\right)}\right)}=\text{Pr}(Q=q=i|U),$$

where $y_{f}^{\left(i\right)}$ is the final output of a KAARMA network trained to recognize the grammar *q* = *i* or classify the word “*i*.” A softmax function is used to ensure that the posterior estimates are non-negative and sum to one. To improve the classification results, we can train several KAARMA networks that specialize in different ordered regions of a word in cascade, as in Figure 7. Since the utterances in the TI-46 digit corpus are not labeled by phoneme, without any frame-to-state alignment computation, we can simply fix the number of grammar states at *L* and partition naively the MFCC sequence for each isolated word into *L* equal segments. When necessary (e.g., total number of frames is less than L), the last MFCC vector is replicated to pad the partition. Each ordered segment is treated as a different grammar state, but given the same class label, and trained using a separate KAARMA network to learn its classification grammar, as shown in Figure 7 (where *L* = 3).

Next, we fix the transition probability for grammar states *q*_*i*_ to *q*_*j*_ in a KAARMA chain at *a*_*i,j*_ = 1, for *j* = *i* + 1, and 0 otherwise. This is a major difference between a standard HMM and a KAARMA chain. The states in an HMM do not cope well with non-stationarity, thus during each Viterbi pass, frame-to-state alignment is performed such that each frame falls into the most likely quasi-stationary region or state in the temporal sequence, and the state transition probabilities are re-estimated. KAARMA and similar recurrent networks, on the other hand, are able to handle non-stationarities by leveraging their internal hidden states **s**_*i*_. One way to visualize these internal hidden states **s**_*i*_ in a grammar state *q*_*i*_ is to view the KAARMA chain as a nested HMM. But unlike the restricted structure of a traditional left-to-right model, the hidden state **s**_*i*_ in each grammar state are free to form transitions that best fit the available data, i.e., an ergodic model, as shown in Figure 7. Finally, in the KAARMA chain formulation, the recognized word is given by the following MAP criterion

(15) $$M^{*}=\underset{M}{\arg\max}\prod_{i=1}^{L}\text{Pr}\left(q_{i}=M|u_{(f\cdot (i-1)/L)+1}^{f\cdot i/L}\right).$$

As discussed earlier, we can further improve the recognition rate by training a second KAARMA network for each grammar state, using the reversed-order feature sequences and multiplying the two softmax scores to derive a bi-directional probability score. By working on smaller segments of a speech signal, not only do we improve the training speed and reduce the need for the KAARMA algorithm to learn long-term dependencies, but also the latency needed for processing sequences of reversed order is shortened.

For real-valued speech features such as MFCCs, we can simply use a Gaussian kernel to apply the KAARMA algorithm for ASR. Next, we describe the appropriate steps for applying the KAARMA chain paradigm to a biologically-inspired ASR system. For each speech signal, biologically-plausible features are generated in the form of spike trains to mimic the front-end filtering performed by the human auditory system.

### 3.2. Spike-based speech representation

Performing adaptive filtering in the RKHS has many advantages. One main merit being that the KAARMA model works with functions in the RKHS transformed by kernels and changing the kernel does not impact the KAARMA algorithm. Therefore, it is agnostic to the type of input and can be applied to any spatiotemporal signal, such as speech, by designing an appropriate kernel. By having separate formulations of the exogenous input vectors **u** and the internal state vectors **s**, the KAARMA algorithm imposes no restriction on the relationship between the two signals in the original input space. We are free to choose the input representations independently as long as the appropriate reproducing kernels are selected. This enables us to work directly with non-numeric bio-inspired data such as spike trains, without modification of the underlying learning algorithm. The theory of RKHS allows signals of heterogeneous types to be operated under a unifying framework in a joint feature space, constructed using either direct sum or tensor-product reproducing kernels.

**Figure 8:**
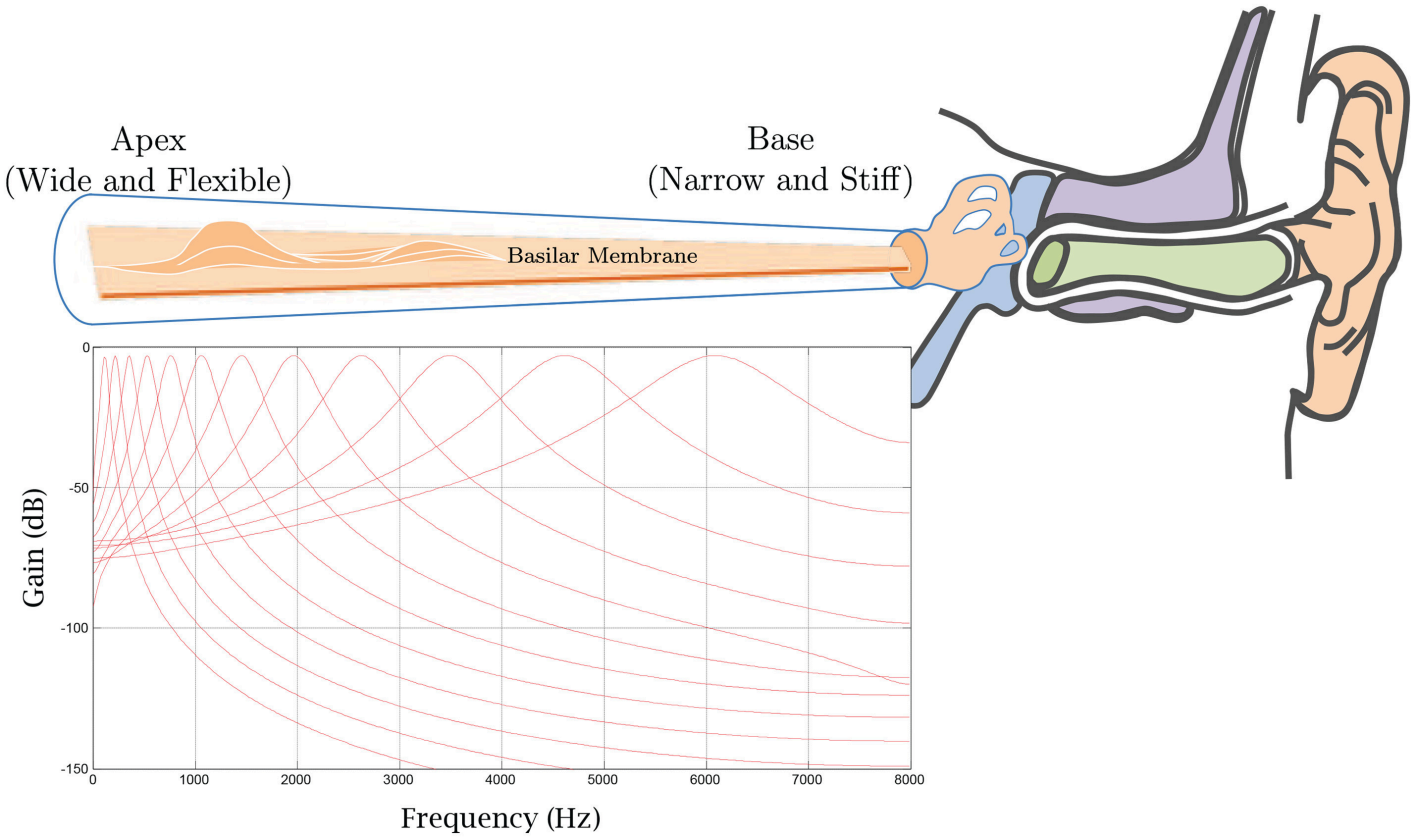
A gammatone filterbank mimics the mechanical response of the cochlea in which the output of each filter models the frequency response of the basilar membrane at a particular location.

**Figure 9:**
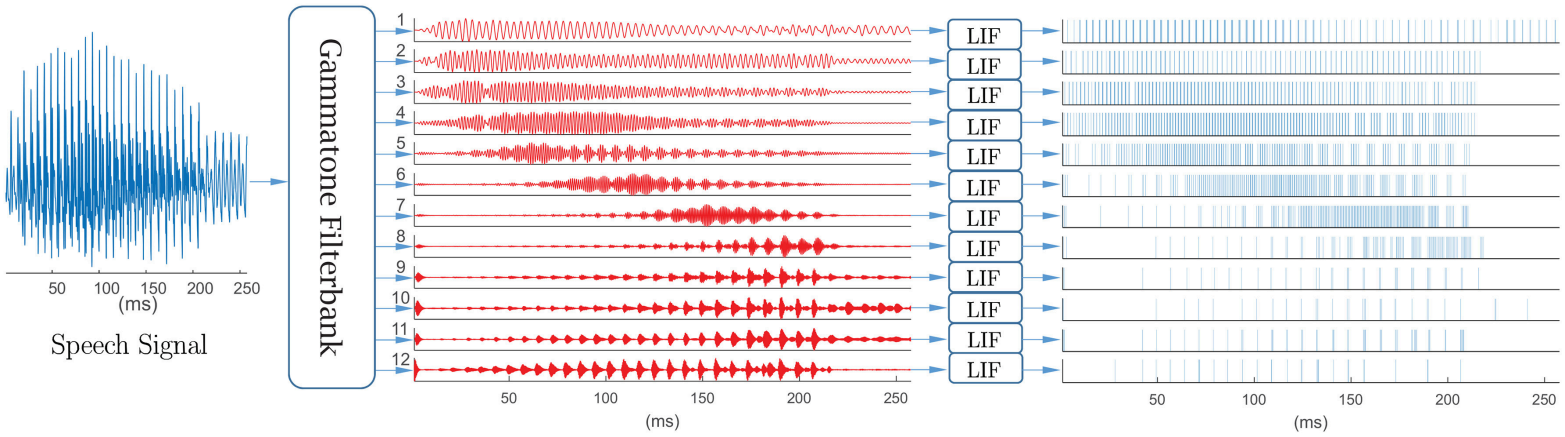
Spike-based front-end for keyword spotting system. Speech signal first passes through a 12-channel output gammatone filterback, with center frequencies equally spaced between 50 Hz and 8 kHz on the ERB-rate scale, then converted into spike trains using leaky integrate-and-fire neurons. The mean spike count per frame (25 ms) ranged from 0.42 to 25.49 and varied across digits and channels.

For our experiments, we combined a gammatone filterbank with a bank of spiking neuron models. First, a gammatone filterbank (Patterson et al., 1987) is applied to each acoustic signal. This formulation is motivated by the mechanical to electrical transduction in the cochlea (Meddis, 1986). Different regions of the basilar membrane vibrate to particular sound frequencies, in response to fluid flow in the cochlea. Sensory hair cells in the organ of Corti then convert the mechanical response to electrical signals which travel along the auditory nerve to the brain for processing. The gammatone filterbank simulates the mechanical response of the cochlea in which the output of each filter models the frequency response of the basilar membrane at a particular location, as shown in Figure 8. Its impulse response is defined in the time domain as

(16) $$g(t)=a_{g}t^{n-1}e^{-2\pi bt}\cos(2\pi f_{c}t+\phi ),$$

where *f*_*c*_ is the center frequency (in Hz), ϕ is the phase of the carrier (in radians), *a*_*g*_ is the amplitude, *n* is the filter order, *b* is the filter bandwidth (in Hz), and *t* indicates time (in s). The output of each gammatone filter is converted into spike trains using LIF neurons with spike-rate adaptation (SRA) and refractory current (Gerstner and Kistler, 2002), as shown in Figure 9.

The LIF neuron captures the basic spiking mechanism of nerve cells and is one of the simplest and most widely used model for spike processing in computational neuroscience. In this biological neuron model, the membrane capacitor *C*_*m*_ is charged by incoming current *I* until its potential *V* exceeds a certain threshold *V*_*th*_, at which time it fires an action potential or spike, discharges, and resets the potential to a level *V*_*reset*_. There are many variants of the model, based on various levels of realism, the one that we will use for this paper is determined by the following resistor-capacitor (RC) equation of the leaky integrator:

(17) $$\tau_{m}\frac{dV}{dt}=(E_{rest}-V)+R_{m}I-E_{sra},$$

where τ_*m*_ = *R*_*m*_*C*_*m*_ is the membrane time constant, *E*_*rest*_ is the resting potential, *R*_*m*_ is the membrane resistance, *I* is the total current flowing into the cell, and instead of a fixed absolute refractory period, a reversal potential for SRA is used and defined as

(18) $$E_{sra}\overset{\Delta }{=}(V-E_{k})R_{m}(g_{sra}+g_{ref}),$$

where *E*_*k*_ is the potassium reversal potential, *g*_*sra*_ and *g*_*ref*_ are the SRA and refractory conductances with time derivatives of *ġ*_*sra*_ = −*g*_*sra*_/τ_*sra*_ and *ġ*_*ref*_ = −*g*_*ref*_/τ_*ref*_, respectively. When membrane potential exceeds the spiking threshold or *V* > *V*_*th*_, SRA and refractory conductances increment by Δ_*sra*_ and Δ_*ref*_ respectively, i.e., the two conductances increase at each spike and decrease exponentially between spikes. Initially, at *t* = 0, we set *V* = *E*_*rest*_.

## 4. Results

As a proof of concept, we used the TI-46 corpus of isolated digits to benchmark the KAARMA-based decoders in this paper. This corpus of speech consists of utterances from 16 English speakers (eight males and eight females) each speaking the digits “zero" through “nine" 26 times. Specifically, 25 out of the 26 utterances were used in the subsequent multispeaker experiments (i.e., our dataset comprises 4,000 of the 4,160 possible utterances). These utterances were further partitioned randomly into a training set (2,700 utterances with an equal number of male/female utterances and digits: 135 utterances per gender, per digit) and a testing set (1,300 utterances with an equal number of male/female utterances and digits: 65 utterances per gender, per digit). Furthermore, to reduce the number of non-speech data points used in the computation and to help align each utterance, speech signals were normalized with respect to their maximum absolute amplitudes, then automatically truncated into the smallest contiguous windows containing all non-silent regions, using a simple threshold-based endpoints detection algorithm.

Next, each truncated utterance was analyzed on 25 ms speech frames at 100 fps. For MFCC front-end, each frame was Hamming windowed, filtered by a first-order pre-emphasis filter (α = 0.95). The magnitude spectrum from the discrete Fourier transform (DFT) was computed and scaled by a Mel-scale triangular filter bank. The output energy was then log-compressed and transformed via the DCT to cepstral coefficients. Thirteen MFCCs were computed per frame, with only the last 12 used as features. In order to highlight the performance difference between context/grammar-based solution delivered by the KAARMA algorithm and results derived from a conventional Markov model, neither the log Parseval energy of each frame nor the time derivatives, i.e., delta and delta-delta coefficients (Furui, 1986), were used as a feature. HMM will benefit from these dynamic spectral features (Skowronski and Harris, 2007). However, our primary goal is to evaluate the performance using a bio-inspired end-to-end spike-based keyword spotting system, without hand-designed feature extraction algorithm past spike generation. The MFCC-HMM design parameters were selected to establish a more comparable baseline without significant increase to complexity.

The performances are summarized in Table 1. The KAARMA solution outperformed the HMM in both the training and testing sets. A big advantage of the KAARMA framework is that it can operate on a single frame at a time, but exploits the full context of an entire input sequence. As a recurrent network, it has an inherent deep structure in time. Furthermore, partitioning each sequence into smaller grammar states improves KAARMA performance and computational efficiency. On the other hand, in general, the amount of data needed to learn an HMM increases quadratically with the number of states.

**Table 1:** Comparisons of KAARMA chain classification accuracies with those of HMMs using an equivalent number of states and a mixture of eight Gaussians per state.

	**Input type**		**Training**	**Testing**
**5-State HMM**				
MFCC			98.74%	98.00 %
Spike train	Rate		93.74%	93.23 %
**5-Network KAARMA Chain**				
MFCC	Sequence ordering:	Left-to-Right	99.33%	98.62
		Bi-Directional	***99.78***%	***99.08***%
Spike train	Rate	Left-to-Right	99.04%	91.85 %
		Bi-Directional	**99.56**%	94.54 %
	Temporal	Left-to-Right	96.70%	93.54 %
	(Spike kernel)	Bi-Directional	98.56%	**95.23** %

*Only 12 MFCC coefficients were used, without log energy and time derivatives. Similarly, only 12 channels of spike trains were used. Bold values indicate the best performance*.

For a comparable processing with the 12 MFCC coefficients used above, to generate the spike trains, a 12-filter gammatone filterbank with center frequencies equally spaced between 50 Hz and 8 kHz on the equivalent rectangular bandwidth (ERB)-rate scale was applied to each acoustic signal. Then, the maximum absolute amplitudes of the 12-channel output were normalized to 4 μ*A* and converted into spike trains using LIF neurons defined by Equation (17). A single neuron is used per channel, for a total of 12 input neurons in this experimental setup. The parameters were membrane resistance *R*_*m*_ = 10 MΩ, time constant τ_*m*_ = 10 ms, spike threshold *V*_*th*_ = −55 mV, spike delta *V*_*spike*_ = 500 mV, reversal potential for SRA *E*_*K*_ = −200 mV, reset potential *V*_*reset*_ = −80 mV, SRA time constant τ_*sra*_ = 200 ms, increase in SRA per spike Δ_*sra*_ = 5 nS, time for refractory conductance to decay τ_*ref*_ = 2 ms, and increase in refractory conductance per spike Δ_*ref*_ = 200 nS. Again, the motivation here is that for a human-engineered speech feature such as MFCC, we can expect reliable performance with only 12 coefficients or inputs. Difference here is that instead of working with waveforms, we encode the information in a sequence of events over time, and not in the amplitude of the signal as is common in ASR. Increasing the number of input channels should improve the recognition accuracy, but as a proof-of-concept, we wanted to evaluate the baseline performance using only 12 channels of spike input.

We directly applied the spike trains in each time frame (temporal coding) as features in our isolated word recognition task. To reduce the bias from data imbalance using the one-vs.-all approach, the positive class (10% of the data for each word model) was replicated three times in the training set with random placement. A five-network KAARMA chain was used to model each word and trained for a single epoch only. To reduce over-fitting, the parameters were not fully optimized over their respective ranges. The results are presented in Table 1. Since HMM does not provide native support for spike trains, the spike count in each frame was used to compute the firing rate and formed a continuous-valued 12-D feature vector across all channels. We also show the five-network KAARMA chain recognition performances using spike-count or rate coding (hidden states **s** ∈ ℝ^3^, kernel parameters *a*_*s*_ = *a*_*u*_ = 5, learning rate η = 0.1, quantization threshold ε = 0.55) and temporal coding (hidden states **s** ∈ ℝ^3^, spike-train kernel parameter *a*_λ_ = 1, hidden-state kernel parameter *a*_*s*_ = 4, learning rate η = 0.1, quantization threshold ε = 0.25) in Table 1.

For rate vectors, a five grammar state KAARMA classifier outperformed similar HMM architecture (five-state with a mixture of eight Gaussians) significantly in the training set, but suffered from overfitting to a greater degree in the testing set. Using temporal coding yields worse performance on the training set, but is better on the test set. This suggests that KAARMA generalizes better using temporal coding of spike trains than rate coding. The information capacity of temporal coding is significantly greater than that of the spike-count rate and is limited only by the temporal resolution of the code. Therefore, the mismatch between model complexity and the task is reduced (spike timing provides additional temporal information over spike count), and the network is less prone to overfitting. On the other hand, spike-count rate is less sensitive to session variability and akin to the spectral power. This is evident from the performances shown in Table 1: left-to-right KAARMA networks can be easily trained to recognize the training set using rate coding (99.04%) vs. temporal coding (96.70%), but the better performance on the test set is given by temporal coding (93.54 vs. 91.85%).

Compared to the left-to-right KAARMA chain test-set performance using MFCCs (98.62%) and that of the HMM (98.00%), in Table 1, we see a drop in accuracies using spike-based front-ends. This is a testament to the popularity of MFCC as the *de facto* speech feature, but also to the fact that the focus of this paper is not to optimize the feature representation, i.e., feature engineering, but rather to demonstrate, as a proof of concept, that a simple spike-based coding scheme achieves competitive result over other ASR systems using spikes.

Furthermore, reversing the input sequence ordering yields a complementary grammar that can be learned using a new set of KAARMA chains, and the two classification outputs can be combined (as discussed in section 3) to enhance recognition accuracy. The results from this formulation are labeled bi-directional in contrast to the natural left-to-right convention. The bi-directional KAARMA spike-based performances are also summarized in Table 1. The best spike test-set performance was given by bi-directional KAARMA chains operating directly on the spike trains (temporal coding) with a recognition accuracy of 95.23% with only one epoch of training.

As noted in a recent publication on LSM-based ASR (Zhang et al., 2015), a systematic comparison with other spike-based methods is difficult. There has been limited research in spike train representations for keyword spotting and speech recognition performances depend largely on specific experimental setups, which often vary greatly and are not fully reported. Most of the recent spike-based ASR systems in the literature utilize a variant of the liquid state machine (Maass et al., 2002). It is interesting to mention that speech was used in this landmark paper as an example of application of LSM, but unfortunately no validation of the method was reported. For a very small subset of the TI-46 corpus of ten different utterances of digits “zero" to “nine” (60% for training and 40% for testing), spoken by five different female speakers, the best LSM achieved a recognition accuracy of 95.5% (Verstraeten et al., 2005). Expanding on the five-speaker result, the state-of-the-art bioinspired performance on a larger subset of the TI-46 digit corpus is reported using a digital LSM (Zhang et al., 2015). For this multispeaker spoken digit task with 1590 speech samples (using five-fold cross validation: 80% used for training and remaining 20% for testing) and training epoch of 500, the final classification rate for the 77-channel spike-input digital LSM is 92.3%. For a smaller subset using a synaptic weight association training (SWAT) SNN, an accuracy of 95.25% was reported (Wade et al., 2010). Our proposed spike-based word spotting system achieved an accuracy of 95.23% for the largest subset with 4,000 samples (67% for training and 33% for testing) and all 16 speakers (eight male, eighht female), using a single training epoch (where only the desired class or 10% of the training data is replicated three times). The results are summarized in Table 2. Again, since the experimental setups are different, the performance comparisons are indicative and not directly quantitative. Nonetheless, spike-input KAARMA achieved over 95% recognition accuracy using the largest subset of the TI46 corpus with the fewest number of spike-train input channels (12) and training epochs (1).

**Table 2:** Comparisons of spike-input KAARMA chain with state-of-the-art SNN and sparse representation on TI46 multispeaker spoken digits.

	**Speakers**	**Samples (Train/Test)**	**Spike train input channels**	**Train epochs**	**Accuracy (%)**
Spike-train KAARMA	16	4,000 $\left(\frac{2}{3}/\frac{1}{3}\right)$	12	1	95.23^†^
Digital LSM (Zhang et al., 2015)	16	1,590 $\left(\frac{4}{5}/\frac{1}{5}\right)$	77	500	92.30
SWAT SNN (Wade et al., 2010)	8	400 $\left(\frac{4}{5}/\frac{1}{5}\right)$	180 input neurons	250	*95.25*
LSM (Verstraeten et al., 2005)	5	500 $\left(\frac{3}{5}/\frac{2}{5}\right)$	39	–	*95.5*

† *Spike-KAARMA achieved over 95% recognition accuracy using the largest subset with the fewest number of input channels and training epochs*.

Furthermore, we note that producing a constant output for time-varying liquid state is a major challenge for LSM, since the memory-less readout has to transform the transient and non-stationary states of the liquid filter into the output without any stable states or attractors to rely on Maass et al. (2002). For the KAARMA formulation using spike-based signals, once the stable dynamics are learned, we can even extract a finite state machine or deterministic finite automata (DFA) from the binary time sequences, where all the information of the input is contained in its temporal evolution, i.e., the inter-spike intervals of individual spike trains, as illustrated in our previous work (Li and Príncipe, 2016).

To further improve the classification accuracies in the current work under clean conditions, we can expand the original feature space by increasing the number of filtered outputs with a larger Gammatone filterbank and corresponding number of LIF neurons. For optimal application-specific results, feature engineering is required to design a set of novel spike-domain attributes.

### 4.1. Computational complexity analysis

For sequence learning (training) of length *n* using KAARMA, where the weight update frequency is only once per sequence, the memory and computation complexities are O(n) and $O\left(n^{2}\right)$, respectively, the same as the simplest online kernel adaptive filter, i.e., the KLMS (Li, 2015). For testing, the memory and computation complexities are O(n), which can be easily implemented using parallel processing in hardware. To further reduce the computational complexity, we use the quantization technique to curb the linear growth of the network by discarding redundant data points and merging the updating coefficients with their nearest neighbors', resulting in a significantly more compact network with size *m* ≪ *n*. The model complexity of KAARMA and other kernel or SVM methods are automatically set by the support vectors, in contrast to neural network based solutions like the SNN. The average number of support or centers of a KAARMA network is 1880.5, compared to the 5,040 neurons in the hidden layer of the SWAT SNN (Wade et al., 2010) and the 135 reservoir neurons in a multilayer 3D grid with thousands of synaptic connections randomly allocated (83 input neurons and 26 readout neurons) of the digital LSM (Zhang et al., 2015). Similarly, we only need to tune a few parameters, compared to the neuron modeling and learning, e.g., spike timing dependent plasticity and Bienenstock-Cooper-Munro learning in Wade et al. (2010). Furthermore, the data requirement to train KAARMA is greatly reduced compared to alternative methods. As shown in Table 2, KAARMA uses orders of magnitude fewer training epochs to converge to a suitable solution.

### 4.2. Noise robustness analysis

We have shown that for clean data, the KAARMA chain solution outperformed the state-of-the-art spike-based ASR system. However, we also see that KAARMA chain operating on spike trains performed worse (for bi-directional sequencing: 95.23 vs. 99.08%) than its MFCC front-end counterpart, for reasons discussed in the above section. A major drawback of MFCC features is their sensitivity to additive noise. Low energy perturbations in the power spectrum are known to cause significant variations after the log compression in their computation (Paliwal, 1998). Spike trains encoded from analogy/digital speech signals using LIF neurons have inherent noise robustness due to the integration or smoothing operation in spike generation.

**Figure 10:**
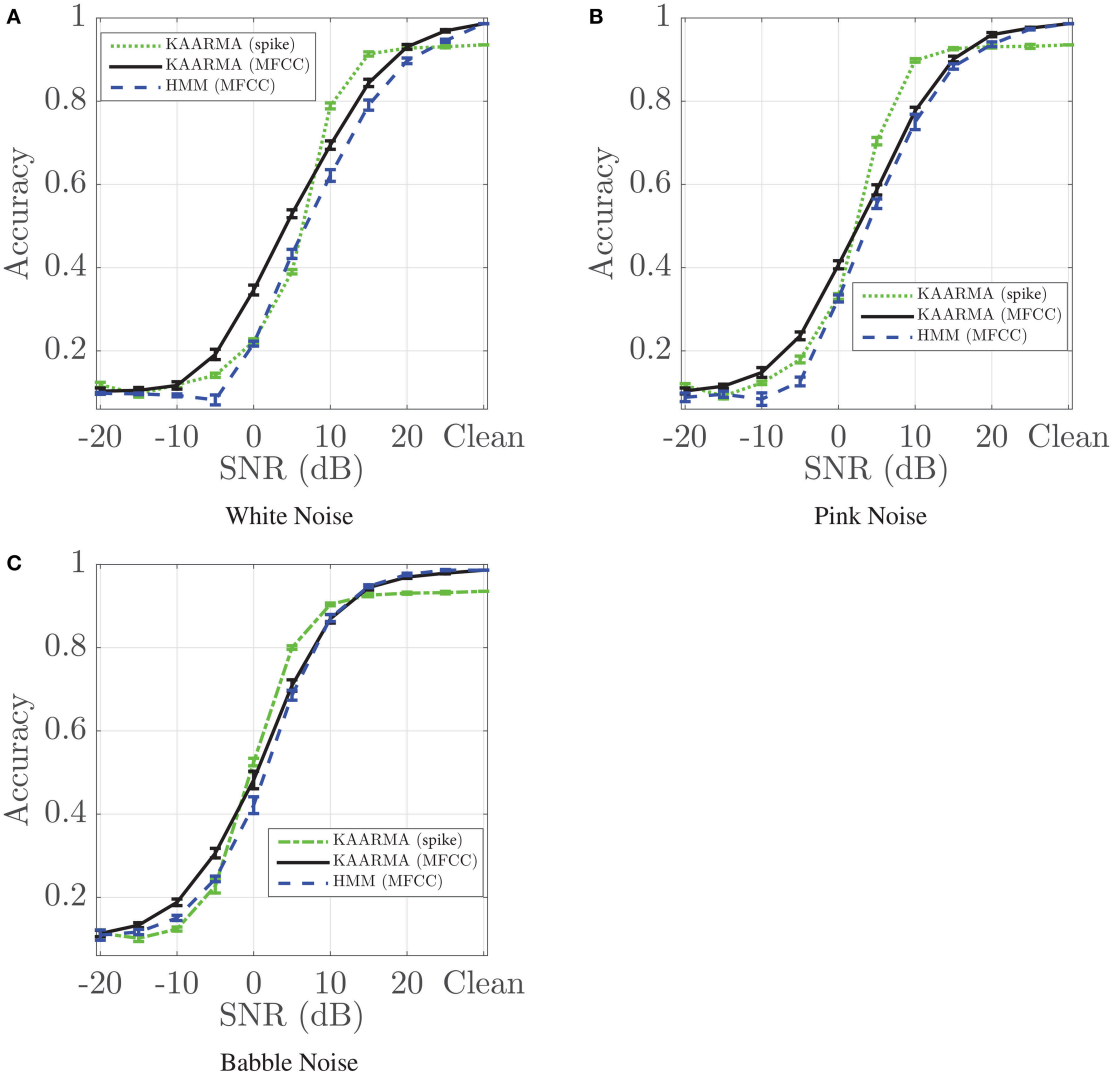
Recognition accuracies for five-network KAARMA chain classifier using spike-train front-end compared with five-network KAARMA chain classifier and HMM using MFCC as a function of SNR. Results (mean ± 1 standard deviation) are averaged over 10 trials with different additive noise. Three types of noise sources are evaluated: **(A)** White noise degrades the performance of Mel-cepstra-based recognition systems most significantly; **(B)** Pink noise is a stationary noise having equal energy per octave; **(C)** Babble noise shares statistical properties of the reference speech and corrupts the entire information bearing spectra. For each noise type, spike-KAARMA classifiers outperformed MFCC-KAARMA and HMM baseline in certain low-SNR regime.

Here we demonstrate that despite this initial performance degradation, KAARMA chain using spike-train front-end shows superior noise robustness in certain low-SNR regime than the MFCC front-end, with three types of noise. Additive white, pink, and multi-speaker babble noise (Hirsch and Pearce, 2000) were introduced to the test utterances, then decoded using the same KAARMA chains trained on noise-free or clean data. Figure 10 shows the classification accuracies of the five-network left-to-right KAARMA chains train using spike-train front-end (green dotted line) as a function of SNR, from −20 to 25 dB in increments of 5 dBs. Again, although the clean-data performance on spike trains is below those of the MFCC-based solutions, the noise robustness is increased with an extended flat region from peak performance, and the drop-off SNR is pushed to the left. In certain low SNR regime, spike-based KAARMA classifiers outperformed five-network KAARMA chains and five-state HMMs using MFCCs. For additive pink noise, we see that KAARMA chain using spike-train front-end outperforms HMM with MFCC for all SNRs below 20 dB. This increased noise robustness demonstrates that neural computation is not merely an artifact of biology, but rather a key to the performance robustness of the auditory system. KAARMA classifiers are able to leverage high-dimensional nonlinear representation of speech in the RKHS, which increases the likelihood of linear class separability in the infinite-dimensional space, and the contextual information provided by the recurrency of the dynamical model.

## 5. Conclusion

We present a biologically-inspired spike-based isolated-word speech recognition or keyword spotting system with superior noise robustness using the KAARMA algorithm. By leveraging the contextual information of the input spike sequence using stable states, KAARMA networks outperform state-of-the-art spike-based processing on the benchmark TI-46 digit corpus. The grammar-based deterministic KAARMA classifier models complex nonlinear dynamical systems using spike train representation and provides a viable alternative to LSMs in small-vocabulary ASR systems and similar applications. By operating in a continuous state space, it has a parsimonious architecture, using hidden states of only three dimensions. Furthermore, spike-based KAARMA classifier outperforms its MFCC counterpart and HMMs in certain low SNR regions.

So far, in this paper, we have only provided a simple spike generation mechanism without any feature engineering step. Speech signals are encoded into spike trains and applied directly to the kernelized recurrent network. In the future, we will investigate ways to optimize the spike-based feature extraction for improved ASR performance, particularly for noisy-data. Specifically, we will address issues such as the number of filters in the gammatone filter-bank and spike-based coding that provides a suitable representation of the local spectral properties in the speech signal.

In earlier works, we represented spike trains as binned binary sequences and trained KAARMA networks to learn the dynamics directly from data, and later extracted the dynamics in the forms of deterministic finite automata (DFA). Computing using DFA is much faster than traditional methods involving analog integration or kernel functions, since state transitions are done automatically based on spike arrival, i.e., a lookup table. We will encode speech spike-train dynamics into DFA in the future. Furthermore, this methodology can be applied to other analog time series, not just limited to speech, using an appropriate analog-to-spike converter. This opens the door to countless novel applications that benefit from improved noise-robustness, ultra-low power, and ultra-fast computation, especially in hardware.

## Author contributions

All authors listed, have made substantial, direct and intellectual contribution to the work, and approved it for publication.

### Conflict of interest statement

We declare a pending patent filed with the University of Florida (UF): Pulse-Based Automatic Speech Recognition, UF #15736; PCT/US2016/065344; WO2017100298A1.

## References

[B1] Bakis R. (1976). Continuous speech word recognition via centi-second acoustic states, in Proc. ASA Meeting (San Washington, DC).

[B2] Baum L. E. Petrie T. Soules G. Weiss N. (1970). A maximization technique occurring in the statistical analysis of probabilistic functions of markov chains. Ann. Math. Stat. 41, 164–171.

[B3] Bengio Y. Ducharme R. Vincent P. Jauvin C. (2003). A neural probabilistic language model. J. Mach. Learn. Res. 3, 137–1155. Aailable online at: http://www.jmlr.org/papers/v3/bengio03a.html

[B4] Bertschinger N. Natschläger T. (2004). Real-time computation at the edge of chaos in recurrent neural networks. Neural Comput. 16, 1413–1436. 10.1162/089976604323057443 15165396

[B5] Boulard H. Morgan N. (1993). Continuous speech recognition by connectionist statistical methods. IEEE Trans. Neural Netw. 4, 893–909. 18276520 10.1109/72.286885

[B6] Bourlard H. Wellekens C. (1990). Links between markov models and multilayer perceptrons, in Proceedings IEEE Transactions on Pattern Analysis and Machine Intelligence Vol. 12, (IEEE) 1167–1178.

[B7] Davis S. Mermelstein P. (1980). Comparison of parametric representations for monosyllabic word recognition in continuously spoken sentences, in Proceedings IEEE Transactions on Acoustics, Speech, and Signal Processing Vol. 28, 357–366.

[B8] Dempster A. P. Laird N. M. Rubin D. B. (1977). Maximum likelihood from incomplete data via the em algorithm. J. R. Stat. Soc. 39, 1–38.

[B9] Dura-Bernal S. Li K. Neymotin S. A. Francis J. T. Principe J. C. Lytton W. W. (2016). Restoring behavior via inverse neurocontroller in a lesioned cortical spiking model driving a virtual arm. Front. Neurosci. 10:28. 10.3389/fnins.2016.00028 26903796 PMC4746359

[B10] Engel Y. Mannor S. Meir R. (2004). The kernel recursive least-squares algorithm. IEEE Trans. Signal Process. 52, 2275–2285. 10.1109/TSP.2004.830985

[B11] Franzini M. A. Lee K. F. Waibel A. (1990). Connectionist viterbi training: a new hybrid method for continuous speech recognition, in Proceedings of International Conference on Acoustics Speech and Signal Processing (Albuquerque, NM), 425–428.

[B12] Frieß T.-T. Harrison R. F. (1999). A kernel based adaline, in ESANN (Bruges), 245–250.

[B13] Furui S. (1986). Speaker-independent isolated word recognition using dynamic features of speech spectrum. IEEE Trans. Acoust. Speech Signal Process. 34, 52–59.

[B14] Gerstner W. Kistler W. M. (2002). Spiking Neuron Models: Single Neurons, Populations, Plasticity. Cambridge, UK: Cambridge University Press.

[B15] Hawkes A. G. (1971). Spectra of some self-exciting and mutually exciting point processes. Biometrika 58, 83–90.

[B16] Haykin S. (1998). Neural Networks: A Comprehensive Foundation, 2nd Edn. Upper Saddle River, NJ: Prentice Hall PTR.

[B17] Hinton G. Deng L. Yu D. Dahl G. E. Mohamed A. Jaitly N. (2012). Deep neural networks for acoustic modeling in speech recognition: the shared views of four research groups. IEEE Signal Process. Mag. 29, 82–97. 10.1109/MSP.2012.2205597

[B18] Hirsch H. G. Pearce D. (2000). The aurora experimental framework for the performance evaluation of speech recognition systems under noise conditions, in Proc. Int. Speech Commun. Assoc. Tutorial Res. Workshop ASR2000 (Paris), 181–188.

[B19] Hopfield J. J. Brody C. D. (2001). What is a moment? Transient synchrony as a collective mechanism for spatiotemporal integration. Proc. Natl. Acad. Sci. U.S.A. 98, 1282–1287. 10.1073/pnas.98.3.1282 11158631 PMC14746

[B20] Jaeger H. (2001). The “Echo State” Approach to Analysing and Training Recurrent Neural Networks. Gmd report 148, German Nat. Res. Cntr. Inf. Technol., Sankt Augustin.

[B21] Kalman R. E. (1960). A new approach to linear filtering and prediction problems. Trans. ASME Ser D. J. Basic Eng. 82, 35–45.

[B22] Kivinen J. Smola A. J. Williamson R. C. (2004). Online learning with kernels. IEEE Trans. Signal Process. 52, 2165–2176. 10.1109/TSP.2004.830991

[B23] Kuhn R. Mori R. D. (1990). A cache-based natural language model for speech recognition. IEEE Trans. Pattern Anal. Mach. Intell. 12, 570–583. 10.1109/34.56193

[B24] Lari K. Young S. J. (1990). The estimation of stochastic contextfree grammars using the inside-outside algorithm. Comput. Speech Lang. 4, 35–56.

[B25] Levin E. (1990). Word recognition using hidden control neural architecture, in Proceedings of the IEEE International Conference on Acoustics, Speech, and Signal Processing (Albuquerque, NM), 433–436.

[B26] Li K. (2015). Adaptive Recurrent Filtering in Reproducing Kernel Hilbert Spaces. Ph.D. dissertation, University of Florida.

[B27] Li K. Ma Y. Príncipe J. C. (2017). Automatic plant identification using stem automata, in 2017 IEEE International Workshop on Machine Learning for Signal Processing (MLSP) (Roppongi).

[B28] Li K. Príncipe J. C. (2016). The kernel adaptive autoregressive-moving-average algorithm. IEEE Trans. Neural Netw. Learn. Syst. 27, 334–346. 10.1109/TNNLS.2015.2418323 25935049

[B29] Li K. Príncipe J. C. (2017a). Automatic insect recognition using optical flight dynamics modeled by kernel adaptive arma network, in 2017 IEEE International Conference on Acoustics, Speech and Signal Processing (ICASSP) (New Orleans, LA), 2726–2730.

[B30] Li K. Príncipe J. C. (2017b). Flight dynamics modeling and recognition using finite state machine for automatic insect recognition, in 2017 International Joint Conference on Neural Networks (IJCNN) (Anchorage, AK), 3733–3740.

[B31] Liu W. Park I. Wang Y. Príncipe J. C. (2009). Extended kernel recursive least squares algorithm. IEEE Trans. Signal Process. 57, 3801–3814. 10.1109/TSP.2009.2022007

[B32] Liu W. Pokharel P. Príncipe J. C. (2008). The kernel least mean square algorithm. IEEE Trans. Signal Process. 56, 543–554. 10.1109/TSP.2007.907881

[B33] Maass W. Legenstein R. A. Bertschinger N. (2005). Methods for estimating the computational power and generalization capability of neural microcircuits, in Advances in Neural Information Processing Systems 17, eds, Saul L. K. Weiss Y. Bottou L. (Vancouver, BC: MIT Press), 865–872.

[B34] Maass W. Natschlager T. Markram H. (2002). Real-time computing without stable states: a new framework for neural computation based on perturbations. Neural Comput. 14, 2531–2560. 10.1162/089976602760407955 12433288

[B35] Meddis R. (1986). Simulation of mechanical to neural transduction in the auditory receptor. J. Acoust. Soc. Amer. 79, 702–711. 2870094 10.1121/1.393460

[B36] Mikolov T. Karafiat M. Burget L. Cernokcy J. Khudanpur S. (2010). Recurrent neural network based language model, in Proceedings of INTERSPEECH (Makuhari), 1045–1048.

[B37] Morgan N. Bourland H. (1990). Continuous speech recognition using multilayer perceptrons with hidden markov models, in Proceedings of the IEEE International Conference on Acoustics, Speech, and Signal Processing (Albuquerque, NM), 413–416.

[B38] Niles L. T. Silverman H. F. (1990). Combining hidden markov models and neural network classifiers, in Proceedings of the IEEE International Conference on Acoustics, Speech, and Signal Processing (Albuquerque, NM), 417–420.

[B39] Paiva A. R. C. Park I. Príncipe J. C. (2009). A reproducing kernel Hilbert space framework for spike train signal processing. Neural Comput. 21, 424–449. 10.1162/neco.2008.09-07-614 19431265

[B40] Paliwal K. K. (1998). Spectral subband centriod features for speech recognition, in Proc. IEEE ICASSP (Seattle, WA), 617–620.

[B41] Park I. M. Seth S. Paiva A. R. C. Li L. Principe J. C. (2013). Kernel methods on spike train space for neuroscience: a tutorial. IEEE Signal Process. Mag. 30, 149–160. 10.1109/MSP.2013.2251072

[B42] Park I. M. Seth S. Rao M. Príncipe J. C. (2012). Strictly positive-definite spike train kernels for point-process divergences. Neural Comput. 24, 2223–2250. 10.1162/NECO_a_00309 22509968

[B43] Patterson R. D. Nimmo-Smith I. Holdsworth J. Rice P. (1987). Annex b of the SVOS final report: an efficient auditory filterbank based on the gammatone function. Appl. Psychol. 1–33.

[B44] Príncipe J. C. Chen B. (2015). Universal approximation with convex optimization: Gimmick or reality. IEEE Comp. Intell. Mag. 10, 68–77. 10.1109/MCI.2015.2405352

[B45] Rabiner L. R. (1989). A tutorial on hidden markov models and selected applications in speech recognition. Proc. IEEE 77, 257–286.

[B46] Ralaivola L. d'Alche Buc F. (2005). Time series filtering, smoothing and learning using the kernel Kalman filter, in IEEE International Joint Conference on Neural Networks, 2005, Vol. 3, (Montreal, QC), 1449–1454.

[B47] Robinson T. (1994). An application of recurrent nets to phone probability estimation. 5, 298–305. 18267798 10.1109/72.279192

[B48] Scholkopf B. Smola A. J. (2001). Learning with Kernels, Support Vector Machines, Regularization, Optimization and Beyond. Cambridge, MA: MIT Press.

[B49] Shpigelman L. Singer Y. Paz R. Vaadia E. (2005). Spikernels: predicting arm movements by embedding population spike rate patterns in inner-product spaces. Neural Comput. 17, 671–690. 10.1162/0899766053019944 15802010

[B50] Skowronski M. D. Harris J. G. (2004). Exploiting independent filter bandwidth of human factor cepstral coefficients in automatic speech recognition. J. Acoust. Soc. Am. 116, 1774–1780. 10.1121/1.1777872 15478444

[B51] Skowronski M. D. Harris J. G. (2007). Noise-robust automatic speech recognition using a predictive echo state network. IEEE Trans Audio Speech Lang. Process. 15, 1724–1730. 10.1109/TASL.2007.896669

[B52] Verstraeten D. Schrauwen B. Campenhout J. V. (2005). Recognition of isolated digits using a liquid state machine, in Proc. SPS-DARTS 2005 (Antwerp), 135–138.

[B53] Viterbi A. J. (1967). Error bounds for convolutional codes and an asymptotically optimal decoding algorithm. IEEE Trans. Inform. Theory 13, 260–269.

[B54] Wade J. J. McDaid L. J. Santos J. A. Sayers H. M. (2010). SWAT: a spiking neural network training algorithm for classification problems. IEEE Trans. Neural Netw. 21, 1817–1830. 10.1109/TNN.2010.2074212 20876015

[B55] Zhang Y. Li P. Jin Y. Choe Y. (2015). A digital liquid state machine with biologically inspired learning and its application to speech recognition. IEEE Trans. Neural Netw. Learn. Syst. 26, 2635–2649. 10.1109/TNNLS.2015.2388544 25643415

[B56] Zhu P. Chen B. Príncipe J. C. (2014). Learning nonlinear generative models of time series with a Kalman filter in RKHS. IEEE Trans. Signal Process. 62, 141–155. 10.1109/TSP.2013.2283842

