# Supplementary material for: Biologically-Inspired Spike-Based Automatic Speech Recognition of Isolated Digits Over a Reproducing Kernel Hilbert Space
Source: Front Neurosci. 2018 Apr 3;12:194. doi: 10.3389/fnins.2018.00194 (PMC5891646; doi:10.3389/fnins.2018.00194)
Supplement: Supplementary file 1 [file Presentation1.pdf]

Supplementary Material: Biologically-Inspired Spike-Based Automatic Speech Recognition of Isolated Digits over a Reproducing Kernel Hilbert Space

Kan Li * **and José C. Príncipe**
*Correspondence:
Kan Li
likan@ufl.edu

1 APPENDIX

The learning algorithm derivation is included here for completeness. Readers are encouraged to refer to (Li and Príncipe, 2016) for a more in-depth discussion.

1.1 Recursive Gradient Descent Learning in the RKHS

In general, the KAARMA algorithm computes the exact error gradient in the RKHS at the end of each input sequence, using the Gaussian or radial basis function kernel

$$\mathcal{K}_a(\mathbf{u}, \mathbf{u}') = \exp\left(-a\|\mathbf{u} - \mathbf{u}'\|^2\right), \tag{S1}$$

with kernel parameter $a > 0$. For hidden state vector $\mathbf{s}$ and input vector $\mathbf{u}$, the joint inner product or tensor-product kernel $\langle \psi(\mathbf{s}, \mathbf{u}), \psi(\mathbf{s}', \mathbf{u}')\rangle_{\mathcal{H}_{su}}$ is computed using $\mathcal{K}_{a_s}(\mathbf{s}, \mathbf{s}')$ and $\mathcal{K}_{a_u}(\mathbf{u}, \mathbf{u}')$, respectively, as

$$\langle \psi(\mathbf{s}, \mathbf{u}), \psi(\mathbf{s}', \mathbf{u}')\rangle_{\mathcal{H}_{su}} = \mathcal{K}_{a_s}(\mathbf{s}, \mathbf{s}') \cdot \mathcal{K}_{a_u}(\mathbf{u}, \mathbf{u}'). \tag{S2}$$

The error gradient with respect to the joint RKHS weights $\mathbf{\Omega}_i$ at time i is

$$\frac{\partial \varepsilon_i}{\partial \mathbf{\Omega}_i} = -\mathbf{e}_i^T \frac{\partial \mathbf{y}_i}{\partial \mathbf{\Omega}_i} = -\mathbf{e}_i^T \frac{\partial \mathbf{y}_i}{\partial \mathbf{s}_i} \frac{\partial \mathbf{s}_i}{\partial \mathbf{\Omega}_i}, \tag{S3}$$

where $\mathbf{e}_i = \mathbf{d}_i - \mathbf{y}_i \in \mathbb{R}^{n_y \times 1}$ is the error vector, $\mathbf{d}_i$ is the desired signal, $\frac{\partial \mathbf{y}_i}{\partial \mathbf{s}_i} = \mathbb{I}$, and the partial derivative $\frac{\partial \mathbf{s}_i}{\partial \mathbf{\Omega}_i}$ consists of n_s state terms, $\frac{\partial \mathbf{s}_i}{\partial \mathbf{\Omega}_i^{(1)}}, \frac{\partial \mathbf{s}_i}{\partial \mathbf{\Omega}_i^{(2)}}, \cdots, \frac{\partial \mathbf{s}_i}{\partial \mathbf{\Omega}_i^{(n_s)}}$. For the k-th state component of $\mathbf{\Omega}_i$, the gradient can be expanded using the product rule as

$$\begin{aligned}\frac{\partial \mathbf{s}_i}{\partial \mathbf{\Omega}_i^{(k)}} &= \frac{\partial \mathbf{\Omega}_i^T \varphi(\mathbf{s}_{i-1}) \otimes \phi(\mathbf{u}_i)}{\partial \mathbf{\Omega}_i^{(k)}} \\ &= \mathbf{\Omega}_i^T \frac{\partial \varphi(\mathbf{s}_{i-1}) \otimes \phi(\mathbf{u}_i)}{\partial \mathbf{\Omega}_i^{(k)}} + \mathbf{I}_{n_s}^{(k)} \left(\varphi(\mathbf{s}_{i-1}) \otimes \phi(\mathbf{u}_i)\right)^T,\end{aligned} \tag{S4}$$

where $\mathbf{I}_{n_s}^{(k)} \in \mathbb{R}^{n_s}$ is the k-th column of the $n_s \times n_s$ identity matrix.

Using the representer theorem, weights $\mathbf{\Omega}_i$ at time i can be written as a linear combination of prior features

$$\mathbf{\Omega}_i = \mathbf{\Psi}_i \mathbf{A}_i, \tag{S5}$$

where $\mathbf{\Psi}_i \triangleq [\varphi(\mathbf{s}_{-1}) \otimes \phi(\mathbf{u}_0), \cdots, \varphi(\mathbf{s}_{m-2}) \otimes \phi(\mathbf{u}_{m-1})] \in \mathbb{R}^{n_\psi \times m}$ is a collection of the m past tensor-product features with potentially infinite dimension n_ψ, and $\mathbf{A}_i \triangleq [\boldsymbol{\alpha}_{i,1}, \cdots, \boldsymbol{\alpha}_{i,n_s}] \in \mathbb{R}^{m \times n_s}$ is the set of corresponding coefficients. Thus, the k-th state component $(1 \leq k \leq n_s)$ of the filter weights matrix in the RKHS at time i becomes

$$\mathbf{\Omega}_i^{(k)} = \mathbf{\Psi}_i \mathbf{A}_i^{(k)} = \mathbf{\Psi}_i \boldsymbol{\alpha}_{i,k}. \tag{S6}$$

Substituting the expression for weights $\mathbf{\Omega}_i$ in (S5) into the feedback gradient on the right-hand side of (S4) and applying the chain rule gives

$$\begin{aligned} \mathbf{\Omega}^T \frac{\partial \psi(\mathbf{s}_{i-1}, \mathbf{u}_i)}{\partial \mathbf{\Omega}^{(k)}} &= \mathbf{A}_i^T \frac{\partial \mathbf{\Psi}_i^T \psi(\mathbf{s}_{i-1}, \mathbf{u}_i)}{\partial \mathbf{s}_{i-1}} \frac{\partial \mathbf{s}_{i-1}}{\partial \mathbf{\Omega}_i^{(k)}} \\ &= \underbrace{2a_s \mathbf{A}_i^T \mathbf{K}_i \mathbf{D}_i^T}_{\boldsymbol{\Lambda}_i} \frac{\partial \mathbf{s}_{i-1}}{\partial \mathbf{\Omega}_i^{(k)}}, \end{aligned} \tag{S7}$$

where $\mathbf{K}_i \triangleq \text{diag}(\mathbf{\Psi}_i^T \varphi(\mathbf{s}_{i-1}) \otimes \phi(\mathbf{u}_i))$ is a diagonal matrix with eigenvalues $\mathbf{K}_i^{(j,j)} = \mathcal{K}_{a_s}(\mathbf{s}_j, \mathbf{s}_{i-1}) \cdot \mathcal{K}_{a_u}(\mathbf{u}_j, \mathbf{u}_i)$ and $\mathbf{D}_i \triangleq [(\mathbf{s}_{-1} - \mathbf{s}_{i-1}), \cdots, (\mathbf{s}_{m-2} - \mathbf{s}_{i-1})]$ is the difference matrix between state centers of the filter and the current input state $\mathbf{s}_{i-1}$. We collect the gradient coefficients in (S7) into a matrix $\boldsymbol{\Lambda}_i \triangleq \frac{\partial \mathbf{s}_i}{\partial \mathbf{s}_{i-1}} = 2a_s \mathbf{A}_i^T \mathbf{K}_i \mathbf{D}_i^T$ which we call the state-transition gradient. Substituting (S7) into (S4) gives the following recursion

$$\frac{\partial \mathbf{s}_i}{\partial \mathbf{\Omega}_i^{(k)}} = \boldsymbol{\Lambda}_i \frac{\partial \mathbf{s}_{i-1}}{\partial \mathbf{\Omega}_i^{(k)}} + \mathbf{I}_{ns}^{(k)} \left(\varphi(\mathbf{s}_{i-1}) \otimes \phi(\mathbf{u}_i)\right)^T. \tag{S8}$$

Again, the states $\mathbf{s}_i$ are assumed hidden and only a final desired output value for $\mathbf{y}_f$ is available at the end of a sequence during training, corresponding to its label. Since (S8) is independent of any teacher signal or error that the system may incur at a future time and can be computed entirely from the observed data, we can forward propagate the state gradients in the recursion. The initial state is user-defined and functionally independent of the filter weights, by setting $\frac{\partial \mathbf{s}_0}{\partial \mathbf{\Omega}_i^{(k)}} = \mathbf{0}$, we can factor out the basis functions and express the recursion as

$$\begin{aligned} \frac{\partial \mathbf{s}_i}{\partial \mathbf{\Omega}_i^{(k)}} &= \boldsymbol{\Lambda}_i \boldsymbol{V}_{i-1}^{(k)} \mathbf{\Psi'}_{i-1}^T + \mathbf{I}_{ns}^{(k)} \left(\varphi(\mathbf{s}_{i-1}) \otimes \phi(\mathbf{u}_i)\right)^T \\ &= \left[\boldsymbol{\Lambda}_i \boldsymbol{V}_{i-1}^{(k)}, \mathbf{I}_{ns}^{(k)}\right] \left[\mathbf{\Psi'}_{i-1}, \varphi(\mathbf{s}_{i-1}) \otimes \phi(\mathbf{u}_i)\right]^T \\ &= \boldsymbol{V}_i^{(k)} \mathbf{\Psi'}_i^T, \end{aligned} \tag{S9}$$

where $\mathbf{\Psi'}_i \triangleq [\mathbf{\Psi'}_{i-1}, \varphi(\mathbf{s}_{i-1}) \otimes \phi(\mathbf{u}_i)] \in \mathbb{R}^{n_\psi \times i}$ are centers generated by the input sequence and forward-propagated states from a fixed filter weight $\mathbf{\Omega}_i$, and $\boldsymbol{V}_i^{(k)} \triangleq \left[\boldsymbol{\Lambda}_i \boldsymbol{V}_{i-1}^{(k)}, \mathbf{I}_{ns}^{(k)}\right] \in \mathbb{R}^{n_s \times i}$ is the updated state-transition gradient, with initializations $\mathbf{\Psi'}_1 = [\psi(\mathbf{s}_0, \mathbf{u}_1)]$ and $\boldsymbol{V}_1^{(k)} = \mathbf{I}_{ns}^{(k)}$.

Updating the weights in the negative direction yields

$$\begin{aligned}
\boldsymbol{\Omega}_{i+1}^{(k)} &= \boldsymbol{\Omega}_{i}^{(k)} + \eta \boldsymbol{\Psi'}_i \left(\mathbb{I}\boldsymbol{V}_i^{(k)}\right)^T \mathbf{e}_i \\
&= [\boldsymbol{\Psi}_i, \boldsymbol{\Psi'}_i] \begin{bmatrix} \mathbf{A}_i^{(k)} \\ \eta \left(\mathbb{I}\boldsymbol{V}_i^{(k)}\right)^T \mathbf{e}_i \end{bmatrix} \\
&\triangleq \boldsymbol{\Psi}_{i+1}\mathbf{A}_{i+1}^{(k)},
\end{aligned} \tag{S10}$$

where η is the learning rate. Since the weights are updated online, to reduce redundancy and better ensure stability, we evaluating each new center from the feature update $\boldsymbol{\Psi}'$ with the existing ones in $\boldsymbol{\Psi}$ using the quantization method outlined in (Li and Príncipe, 2016, Algorithm 2).

REFERENCES

Li, K. and Príncipe, J. C. (2016). The kernel adaptive autoregressive-moving-average algorithm. *IEEE Trans. Neural Netw. Learn. Syst.* 27, 334–346
